# *Chlamydia trachomatis In Vivo* to *In Vitro* Transition Reveals Mechanisms of Phase Variation and Down-Regulation of Virulence Factors

**DOI:** 10.1371/journal.pone.0133420

**Published:** 2015-07-24

**Authors:** Vítor Borges, Miguel Pinheiro, Minia Antelo, Daniel A. Sampaio, Luís Vieira, Rita Ferreira, Alexandra Nunes, Filipe Almeida, Luís J. Mota, Maria J. Borrego, João P. Gomes

**Affiliations:** 1 Reference Laboratory of Bacterial Sexually Transmitted Infections, Department of Infectious Diseases, National Institute of Health, Lisbon, Portugal; 2 Bioinformatics Unit, Department of Infectious Diseases, National Institute of Health, Lisbon, Portugal; 3 School of Medicine, University of St Andrews, St Andrews, Scotland, United Kingdom; 4 Innovation and Technology Unit, Human Genetics Department, National Institute of Health, Lisbon, Portugal; 5 UCIBIO, REQUIMTE, Departamento de Ciências da Vida, Faculdade de Ciências e Tecnologia, Universidade Nova de Lisboa, Caparica, Portugal; University of the Pacific, UNITED STATES

## Abstract

Research on the obligate intracellular bacterium *Chlamydia trachomatis* demands culture in cell-lines, but the adaptive process behind the *in vivo* to *in vitro* transition is not understood. We assessed the genomic and transcriptomic dynamics underlying *C*. *trachomatis in vitro* adaptation of strains representing the three disease groups (ocular, epithelial-genital and lymphogranuloma venereum) propagated in epithelial cells over multiple passages. We found genetic features potentially underlying phase variation mechanisms mediating the regulation of a lipid A biosynthesis enzyme (CT533/LpxC), and the functionality of the cytotoxin (CT166) through an ON/OFF mechanism. We detected inactivating mutations in CT713/*porB*, a scenario suggesting metabolic adaptation to the available carbon source. CT135 was inactivated in a tropism-specific manner, with CT135-negative clones emerging for all epithelial-genital populations (but not for LGV and ocular populations) and rapidly increasing in frequency (~23% mutants *per *10 passages). RNA-sequencing analyses revealed that a deletion event involving CT135 impacted the expression of multiple virulence factors, namely effectors known to play a role in the *C*. *trachomatis* host-cell invasion or subversion (e.g., CT456/Tarp, CT694, CT875/TepP and CT868/ChlaDub1). This reflects a scenario of attenuation of *C*. *trachomatis* virulence *in vitro*, which may take place independently or in a cumulative fashion with the also observed down-regulation of plasmid-related virulence factors. This issue may be relevant on behalf of the recent advances in *Chlamydia* mutagenesis and transformation where culture propagation for selecting mutants/transformants is mandatory. Finally, there was an increase in the growth rate for all strains, reflecting gradual fitness enhancement over time. In general, these data shed light on the adaptive process underlying the *C*. *trachomatis in vivo* to *in vitro* transition, and indicates that it would be prudent to restrict culture propagation to minimal passages and check the status of the CT135 genotype in order to avoid the selection of CT135-negative mutants, likely originating less virulent strains.

## Introduction

Experimental evolution studies have exploited several organisms’ traits to obtain a real-time window on evolutionary processes, to reveal new genetic targets of selection and, in general, to get insight on the genome-scale dynamics of natural selection within microbial populations, constituting a rare opportunity to deeply uncover the correlation (or lack of such) between genetic and phenotypic signatures [1–3]. In bacteria, common findings include the detection of genes or mutations underlying phenotypic alterations typically observed in laboratory evolving bacterial populations, such as: evolution of new (or loss of) metabolic capabilities, development of antibiotic resistance or sensitivity, or loss of virulence (namely, reduction of the infective capacity) [1, 3–9]. For the latter alteration, it has been also observed that: *i*) *in vitro*-cultured microorganisms are in general less virulent than the corresponding wild-types; *ii*) virulence decreases gradually during *in vitro* culture; and *iii*) virulence can eventually be restored by *in vivo* passage [10–14]. These features have consequently been explored by researchers in order to enlighten pathogenic mechanisms underlying the process of infection, and also uncover new virulence genes. Indeed, loss of function of virulence factors may be possibly related to their dispensability in the *in vitro* environment, reinforcing the assumption that these genes may play an essential and specific role *in vivo*.

Few studies have evaluated the impact of laboratory propagation on the population dynamics of obligate intracellular bacteria [15–19], wherein the evolutionary process is ruled by the interaction with the host. In this regard, the use of laboratory-passaged strains has been frequently questioned when studying *Chlamydia*, including the major human pathogen *C*. *trachomatis* [17, 19, 20–24], which are organisms displaying a highly specialized capacity of adaptation to the intracellular life-style. Indeed, these obligate intracellular bacteria have a unique biphasic developmental cycle alternating between an infectious form, the elementary body (EB), and a noninfectious replicating form, the reticulate body (RB). Shortly after entering the target eukaryotic cells, EBs differentiate into RBs, which replicate within a membrane-bound parasitophorous vacuole termed the “inclusion”, until the newly formed infectious EBs are released to infect neighboring cells. *C*. *trachomatis* exploits the host cell throughout all developmental cycle by subverting critical cellular functions (such as, cytokinesis, apoptosis, host cytoskeleton, nutrient transport, membrane trafficking pathways or immune responses) [25–28] essentially by effector proteins translocated into the host cytosol or localized into the inclusion membrane (Inc proteins) [29–31]. The majority of the *C*. *trachomatis* effectors and Incs described so far were found to be transported by using a type III secretion (T3S) system [29, 31–38]. Despite the high degree of genomic conservation in both the 1Mb chromosome and the virulence regulator 7.5 kb plasmid, strains from different *C*. *trachomatis* serovars typically present dissimilar tissue tropism, disease outcomes and clinical prevalence. In fact, serovars A-C are normally associated with ocular infections leading to blinding trachoma, strains from serovars D-K are the major cause of bacterial sexually transmitted infections (where serovar E is the most clinically prevalent followed by F), and strains from serovars L1-L3 are the causative agents of typical bubonic lymphogranuloma venereum (LGV) or the LGV-associated proctitis [39–41] (the latter is primarily caused by L2b strains [42]).

In a previous approach to study the *in vitro* population dynamics for this bacterium [15], we observed that culture propagation yielded discreet genomic changes in the absence of specific selective pressures. Despite the advance of culture-independent *C*. *trachomatis* genome sequencing [22, 43–45], our data suggest that the *in vivo*-derived genetic make-up is not strongly compromised during the classical culture procedures for propagating *C*. *trachomatis*. However, this discreet scenario of genomic alterations may strongly impact gene expression and virulence. Indeed, we detected that clones carrying an inactivated version of the recently identified virulence gene CT135 (which is believed to function in sustaining a more persistent or chronic infections *in vivo* [46, 47]), became 100% frequent in the population of an epithelial-genital strain [15]. Furthermore, assessing the impact of introducing *C*. *trachomatis in vitro* gains special relevance in light of the recent rampant development of transformation and gene knockout experiments in *C*. *trachomatis*, which demand culture propagation [48–52]. Thus, it is crucial to well characterize the *C*. *trachomatis in vivo* to *in vitro* transition process in order to avoid bias in the interpretation of downstream mutagenesis-derived data and also because virulence of propagated strains may be attenuated. We now performed an experimental evolution study over *C*. *trachomatis* strains from serovars displaying dissimilar clinical prevalence and representing the three disease groups. We evaluated the dynamics by which mutations appear and their frequency in the evolving bacterial populations through whole-population sequencing at various time-points after *C*. *trachomatis* introduction *in vitro*. We also investigated transcriptome alterations throughout adaptation. Specifically, we aimed to answer the following questions concerning the *in vitro* adaptation of *C*. *trachomatis*: *i*) is the duration of bacterial developmental cycle affected?; *ii*) is the adaptive dynamics dependent on the tissue-specificity or disease outcomes of the infecting strains?; *iii*) which are the genomic and transcriptomic events underlying adaptation?; and *iv*) is there parallelism in the adaptive outcomes across strains?

## Materials and Methods

### *Chlamydia trachomatis* strains

Six *C*. *trachomatis* strains belonging to the three disease groups (one ocular strain, four epithelial-genital strains and one strain associated with LGV-proctitis) were used in the present study. The *ompA* genotype was verified for all strains, as previously described [53]. The ocular strain C/TW-3 was isolated from the human conjunctiva and was obtained from the American Type Culture Collection (ATCC VR-1477). The remaining strains belong to the culture collection of the Portuguese National Institute of Health. The epithelial-genital clinical isolate Ia/CS190/96 was isolated from a male urethra, whereas the epithelial-genital D/CS637/11, F/CS847/08 and E/CS1025/11 were collected from swabs of the cervix of women with no symptoms, HPV-associated warts, and no available clinical data, respectively. Finally, the LGV strain L2b/CS19/08 was isolated from a anorectal swab of a 29-years old HIV(+) man with proctitis and syphilis.

### *C*. *trachomatis in vitro* propagation and preparation for whole-population full-genome sequencing

Each clinical specimen (swabs) in transport medium (2 sucrose phosphate buffer supplemented with gentamicin, vancomycin and nistatin) or the ATCC inocula (for the C/TW-3 strain) was inoculated in HeLa229 confluent monolayers (cultured on 24-well plates in MEM containing 10% fetal bovine serum, 5 mM L-glutamic acid, 10 μg/ml gentamicin and 0.5 μg/ml fungizone at 37°C, 5% CO_2_) by centrifuging at 2110 rcf for 1h at 34°C [54]. The cultures were then incubated for 1h at 37°C, 5% CO_2_, and the cell medium was subsequently replaced by fresh medium supplemented with vitamins (1x), non-essential aminoacids (1x), glucose (0.5%) and cycloheximide (0.5 μg/ml) (so called “enriched medium”). Cultures were allowed to grow at 37°C, 5% CO_2_. At the end of the bacterial developmental cycle, the infected cells were harvested using glass beads and disrupted through sonication (Vibra Cell, Bioblock Scientific), and further submitted to low-speed centrifugation (100 rcf for 7min). The bacterial-enriched supernatant was collected and re-inoculated by centrifugation (835 rcf for 1h) onto new HeLa229 confluent monolayers (first passage). Each chlamydial culture was continuously passaged in T25 cm^2^ flasks, so the passage numbers for the clinical isolates refer to the total number of passages after inoculating the content of the clinical swab. Inoculations were always preceded by sonication followed by low-speed centrifugation, so that the bacterial life-cycle was continuously synchronized. The volume of transferred inocula ranged from 1/10 to 1/2 of the total suspension of the previous passage (depending on the bacterial-enrichment), limiting drift and random selection of clones derived from bottlenecks. Strains D/CS637/11, E/CS1025/11, F/CS847/08 and L2b/CS19/08 were propagated over 30 *in vitro* passages, whereas strains Ia/CS190/96 and C/TW-3 were propagated until the passage 100. The propagation extension of these two strains relied on the need either to clarify the evolutionary scenario that was being observed (for the former) or to obtain a long-term adaptive landscape for an ocular strain (for the latter), mirroring previous data for one LGV and one epithelial-genital strain [15]. Passages ~6, 20, 30, 50 and 100 were selected for whole-population full-genome sequencing (see below), where culture scale-up to obtain enough DNA for sequencing was performed two or three passages before each time-point. The contents of these flasks was harvested and further subjected to a discontinuous density gradient purification procedure, before proceeding with DNA extraction and quantification as previously described [15].

### Whole-population full-genome sequencing

DNA samples were used to prepare Illumina paired-end libraries according to the manufacturer's instructions (Illumina Inc., San Diego, CA). Libraries were subsequently subjected to cluster generation and paired-end sequencing (2x250 bp) by using the next-generation sequencing platform Illumina MiSeq (Illumina Inc., San Diego, CA, USA), according to the manufacturer’s instructions. FastQC (http://www.bioinformatics.babraham.ac.uk/projects/fastqc/) and FASTX (http://hannonlab.cshl.edu/fastx_toolkit/) tools were applied to check and improve the quality of the raw sequence data, respectively. Reads were mapped to *C*. *trachomatis* chromosome and plasmid sequences available at GenBank using both Bowtie2 [version 2.1.0 (http://bowtie-bio.sourceforge.net/bowtie2/index.shtml)] [55]. SAMtools/BCFtools (http://samtools.sourceforge.net/) [56] were applied to call single nucleotide polymorphism (SNPs) and insertions/deletions (indels), which were carefully confirmed through visual inspection using the Integrative Genomics Viewer [version 2.3.12; (http://www.broadinstitute.org/igv)] [57]. The assembled chromosome and plasmid sequences obtained from the first time-point were used to map the reads generated from subsequent time-points. The frequency of the mutations in the population at the time of sampling were carefully scrutinized through IGV inspection. The mutations reported were supported by a depth of coverage above 150x, a score of quality above 20 for more than 90% of the bases sustaining each polymorphism and a relative percentage of reads mapping to either of the two DNA strands (strand bias) ranging from 40 to 60%. We also inferred the plasmid copy number *per* chromosome throughout the different time-points, based on the ratio plasmid/chromosome taken from the respective depth coverage. Chromosome and plasmid sequences obtained at the first time-point were annotated by the NCBI Prokaryotic Genomes Annotation Pipeline 2.3 and deposited in GenBank (see accession numbers in the S1 Table). For the global evaluation of the whole genomic backbone of the studied *C*. *trachomatis* genomes, we compared them with the ones from 52 previously sequenced *C*. *trachomatis* strains [58], as described elsewhere [59].

### Impact of *in vitro* passaging on the *C*. *trachomatis* growth rate

The growth rate (and doubling times) of bacterial populations was measured at the initial and final stages of the evolution experiment in order to assess the impact of *in vitro* propagation on the bacterial fitness (i.e., the ratio of growth rates of evolved population relative to its ancestral). Briefly, for each bacterial population, HeLa229 confluent monolayers on 6-well plates were inoculated (by centrifuging at 2110 rcf for 1h at 34°C) at a multiplicity of infection of about 1 with fresh inocula obtained from the cultures (in T25 cm^2^ flasks) under passaging. Cells were harvested for DNA extraction at 4, 10, 20 hours post-infection and at the end of the bacterial life-cycle (ensuring that cell lysis had not occurred). DNA samples were then used to quantify the number of *C*. *trachomatis* genomes at each time-point through real-time quantitative PCR (qPCR), as previously described [15]. The growth rate and doubling times (dt) were calculated using the standard formulas: growth rate = (*ln* N_1_ –*ln* N_0_) / t _(1–0)_ and dt = (t_1_ –t_0_)/n, where (t_1_—t_0_) is the time elapsed during the period of higher growth and n is the number of bacterial generations. The n was calculated based on the formula: n = (log_10_N_1_/N_0_)/log_10_2, where N_1_ and N_0_ are the genome copy numbers determined at the defined time-points.

### Yield of infectious progeny of the serovar D CT135-positive and CT135-negative isolates in cell culture

In order to complement the data from growth rate assays and to obtain an alternative perspective of the impact of the *in vitro* propagation on the chlamydial replicating capacity, we further evaluated the yields of inclusion-forming units (IFUs) (as a measure of progeny) of the serovar D clinical isolate at beginning of the experiment (here designated as “CT135-positive”) and after CT135 loss (here designated solely as “CT135-negative”, although the two flanking genes were also partially deleted). We grew both the D/CS637/11 CT135-positive and CT135-negative strains in HeLa229 cells, and used serial dilution titration to determine the IFU *per* milliliter of the EB-enriched stocks. Briefly, serial dilutions were inoculated as above in HeLa229 monolayers seeded on 96-well plates, the cells were fixed at 22 h post-infection with methanol, stained with an anti–*C*. *trachomatis* lipopolysaccharide antibody (Pathfinder), and the inclusions were enumerated by immunofluorescence microscopy. Inocula of equivalent concentration (IFU *per* militer) for both the ancestral and the evolved strain were then applied to infect HeLa229 monolayers (on 6-well plates) at a MOI of 1. At the time-points 6, 12, 18, 24, 30, 36, 42 and 48 post-infection, the cultures were harvested and followed the same procedure of serial dilution titration for inclusion counting and further construction of one-step growth curves. These curves were also important to define the proper time-points to be used in transcriptome (RNA-seq) assays.

### Evaluation of molecular stability of CT135 transcripts

The molecular stability of the bacterial transcripts is traditionally evaluated throughout the quantification of the mRNA half-life time after transcriptional blockage with the antibiotic rifampicin [60, 61], which inhibits the *de novo* synthesis of RNA. Since the quick degradation of mRNAs from genes that are deleterious in a given environment is believed to confer selective evolutionary advantage [62], we sought to evaluate the molecular stability of CT135 transcripts before and after the spread of CT135-negative clones. We performed this assay for the clinical isolates with emergent CT135-negative clones. After a preliminary optimization stage, we opted to use a final rifampicin concentration of 10 μg/ml (Sigma-Aldrich) since it allowed a maximum bacterial transcriptional arrest while causing no visible morphologic effects on HeLa229 cells (data not shown). This concentration is in agreement with the one used in a previous study aiming to assess the molecular stability of transcripts in other *Chlamydia* species [61]. For the assays to determine CT135 mRNA half-life time, fresh EB-enriched inocula from each isolate under passaging (for both ancestral and evolved populations) were inoculated as above on 6-well plates, and cultures were allowed to grow until antibiotic treatment. Considering that higher levels of CT135 transcripts are detected at early stages of the *C*. *trachomatis* life-cycle [32, 63], we opted to evaluate the molecular stability of the CT135 transcripts at this stage. Hence, at 4h pi, the culture medium was replaced by fresh medium containing rifampicin and after 10 min cells were scraped with PBS and mechanically disrupted by sonication. The final suspension was rigorously divided into two identical aliquots and immediately frozen in liquid N_2_. Culture samples were similarly harvested before rifampicin addition (at 4h pi). The twin aliquots were submitted to independent DNA and RNA extraction using QIAamp DNA Mini Kit (Qiagen) and RNeasy Mini Kit (Qiagen), respectively, according to manufacturer's instructions. Purified RNA samples were further subjected to cDNA generation using TaqMan Reverse Transcription (RT) Reagents (Applied Biosystems, Life Technologies, Branchburg, NJ, USA), as previously described [64]. cDNA samples were used to quantify the transcript’s level through qPCR before and after rifampicin treatment. In order to overcome putative bias associated with differential degradation along the operon, we applied two independent qPCRs targeting the two genes (CT134 and CT135) [46], and calculated a mean value. The mRNA levels were normalized against the number of *C*. *trachomatis* genomes quantified on the corresponding DNA samples, using a previously described qPCR [65]. All quantifications were performed using a LightCycler 480 (LC480) Instrument (Roche). The reagents consisted of LightCycler 480 SYBR Green I Master (Roche), 400 nM of each primer and 5 μl of sample DNA or cDNA, in a final volume of 25 μl. The sequences of all primers used in this study are listed in the S2 Table. The mRNA half-life times (t ½) were calculated by using an adaptation of the “two-fold” decay step method [66] based on the fit of an exponential decay between values obtained at the first time-point (before rifampicin addition) (N_0_) and the values calculated *t* minutes (10 min) after the transcriptional arrest (N_1_), using the formula: t ½ = -ln2/*k*; where the rate of decay rate (*k*) was estimated as follows: *k = ln(N*_*1*_ */N*_*0*_ *)/ t*.

### Evaluation of the impact of a deletion event involving CT135 on *C*. *trachomatis* transcriptome

Analyses of global gene expression in *C*. *trachomatis* through microarrays or RNA-seq have been almost exclusively performed at mid-stage of the developmental cycle in order to ensure the detection of transcripts from a higher number of genes [51, 63, 67–69]. Here, we also focused on the bacterial mid-cycle, and chose the time-point for RNA-seq differential expression analysis according to the distinct growth dynamics observed for the D/CS637/11 CT135-positive and CT135-negative isolates (evaluated by IFU-based growth curves) (see Results section). Hence, HeLa229 confluent monolayers (cultured on T25 cm^2^ flasks) were infected with serovar D CT135-positive (passage 7) and CT135-negative (passage 20) isolates at a MOI of about 1 (as described above), and were grown for 30 h and 24 h post-infection, respectively. Subsequently, the culture medium was discarded, cells were harvested using 2:1 volumes of RNAprotect bacteria reagent (Qiagen, Valencia, CA), sonicated, and subsequently centrifuged at 100 rcf for 7 min. The supernatant was collected, pelleted by centrifugation (18500 rcf for 15 min) and further processed for RNA extraction using the RNeasy Mini Kit (Qiagen), according to manufacturer's instructions. Samples were treated on column with RNase-free DNase (Qiagen), and RNA samples were subjected to Agilent Bioanalyser (Agilent Technologies) analyses before and after the bacterial mRNA enrichment using MICROBEnrich (Ambion, Austin, Texas) and MICROBExpress (Ambion) protocols, according to manufacturer’s instructions. Bacterial mRNA-enriched samples were further subjected to library construction (TruSeq Stranded mRNA sample preparation kit, Illumina) and sequencing on an Illumina MiSeq sequencer using a paired-end (2x150bp) strategy (about 15M reads were dedicated *per* sample). The quality of the raw sequence data was assessed through FastQC analysis. The sequence reads were mapped (using Bowtie2) to the D/CS637/11 genome (sequenced in this study) and the expression of each chromosome and plasmid coding sequence (CDS) was normalized as fragments *per* kilobase of CDS *per* million mapped reads (FPKM) using the Cufflinks (version 2.1.1; http://cufflinks.cbcb.umd.edu/). In order to overcome the variable efficiency of the mRNA enrichment step across samples and to improve the overall robustness of transcript abundance estimates, reads from bacterial rRNA and tRNA were selectively masked in the analyses. Differential gene expression analyses between D/CT135-positive and D/CT135-negative strains were conducted using Cuffdiff, where FPKMs and fragment counts are scaled taking the median of the geometric means of fragment counts across all libraries (geometric normalization method). This rationale is identical to the one used by DEseq [70]. The Benjamini–Hochberg procedure, which controls the false discovery rate (FDR), was applied to adjust raw *P-*values for multiple testing [71]. Genes were considered to be differentially expressed when the fold change of expression exceeds the two-fold and the adjusted *P*-values were <0.05. RT-qPCR validation of the RNA-seq results was performed on a LC480 apparatus (as described above) using cDNA samples obtained from total RNA aliquots collected before depletion of rRNA and polyadenylated mRNAs. RT-qPCR data for each gene were normalized against the data obtained for the 16S rRNA transcript. Differential expression data are based on two biological replicates. In order to ensure that the observed expression differences were due specifically to the deletion event involving CT135 and not to other factors underlying laboratory propagation, we performed a control RNA-seq experiment by analysing the Ia/CS190/96 isolate within the same time frame as for the serovar D strain. This shows up as a suitable control, since this strain did not reveal any mutation in the entire genome throughout the same passaging period (i.e., until passage 20).

## Results

### The genome make-up of studied strains represents the major branches of the species tree

We performed experimental evolution of *C*. *trachomatis* ocular (C/TW-3; an historical prototype strain), epithelial-genital (D/CS637/11, E/CS1025/11, F/CS847/08 and Ia/CS190/96; clinical isolates) and LGV (L2b/CS19/08; an anorectal isolate associated with LGV-proctitis) strains. Among these, we recently described the genomes of C/TW-3 [72] and L2b/CS19/08 [54]. To integrate the genetic backbone of all strains used in this work in the frame of the known species phylogeny and diversity [58], we first determined and analyzed the genome of the strains after minimal passages. We observed the phylogenetic segregation of each genome within the four major branches of the species tree, where each strain clustered with the other *C*. *trachomatis* strains sharing not only the tissue-specificity but also the clinical prevalence of the corresponding serovars (S1 Fig). Of note, two strains (Ia/CS190/96 and F/CS847/08) revealed clear traces of recombination involving epithelial-genital strains (S1 Fig), similarly to what has been extensively described [58, 73, 74]. Globally, the present study aiming to evaluate the dynamics underlying the *in vivo* to *in vitro* transition of *C*. *trachomatis* involves strains representing, not only the three disease groups (ocular, epithelial-genital and LGV), but also the four major genetic branches of the species tree.

### Initial populations reveal some degree of allelic variation

**Fig 1:**
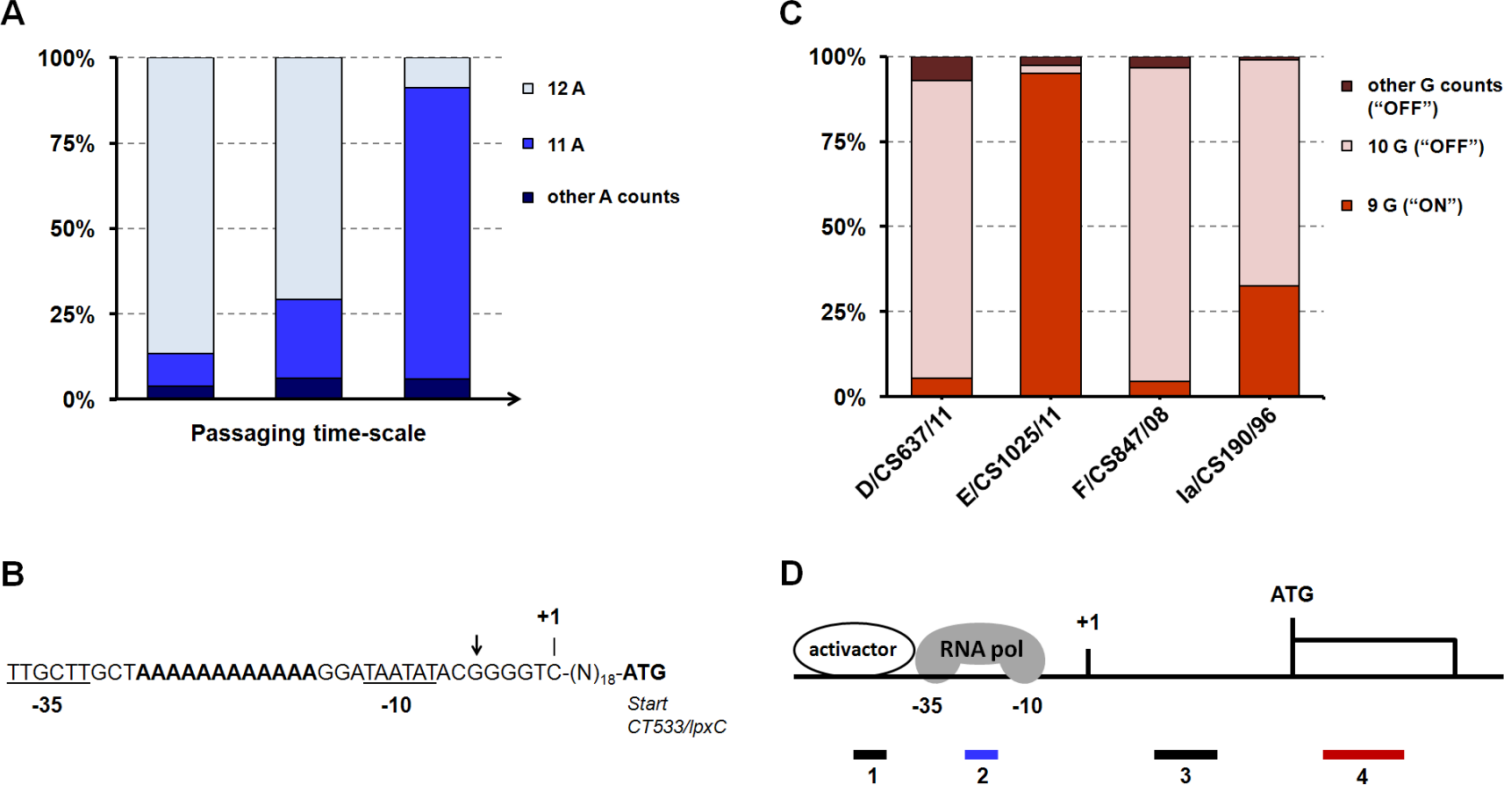
Phase variation mediated by variable homopolymeric tracts. Panel A. The graph shows the evolution throughout passaging of the percentage of sequence reads with different ‘A’ counts in the homopolymeric tract upstream from CT533/*lpxC* for the strain E/CS1025/11. The poly(A) tract corresponds to poly(T) in the annotated leading strand. Panel B. Schematic view of the putative promoter region of CT533/*lpxC*. The predicted transcription start site [126] is labeled by +1. The variable poly(A) tract (in bold) falls between the predicted -35 and -10 hexamers (underlined). BLAST analyses revealed the existence of variable number of ‘A’ counts in *C*. *trachomatis* genomes, and also that the nucleotide indicated with an arrow is deleted exclusively in all LGV strains. Panel C. The graph shows the percentage of sequence reads with different ‘G’ counts in the variable homopolymeric tract of CT166 found in the initial populations of the epithelial-genital strains. “G” counts of nine correspond to an “ON” protein. Panel D. Schematic view of the four positions (numbers 1 to 4) relative to a gene at which contingency loci (e.g., homopolymeric tracts) can cause phase variation (adapted from van der Woude MW and Bäumler AJ, Clin Microbiol Rev **17**:581–611, 2004 [152]). Whereas positions 1 and 2 are associated with transcription initiation and position 4 with translation (ON/OFF), the mechanism regarding the position 3 is not completely disclosed. We found heterogeneity in length within homopolymeric tracts located in positions 2 (for CT533/*lpxC*) (blue), 3 (for CT043/*slc1* and the operon CT134-CT135), and 4 (for CT166—cytotoxin) (red).

Genomic analyses of the initial populations revealed phenomena associated with heterogeneity within DNA homopolymeric tracts located in the gene encoding the cytotoxin (CT166) and upstream from the gene CT533/*lpxC*, which encodes an essential enzyme [UDP-3-O-(R-3-hydroxymyristoyl)-GlcNAc deacetylase] in the biosynthesis of lipid A [75, 76]. Cytotoxin will be focused on the next section as the detected variability may underlie its functionality. Regarding the CT533/*lpxC*, for strain E/CS1025/11 we found a homopolymeric tract 36 bp upstream from the start codon (between the predicted -35 and -10 TATA boxes) with a string of 12 ‘A’ in 87% of the reads and 11 ‘A’ in 9% of the reads (4% for other ‘A’ counts) (Fig 1A and 1B). Although a similar scenario was also observed for initial populations of the other strains, we drew attention to E/CS1025/11, since we detected a progressive increment of the 11 poly(A) tract throughout bacterial propagation (Fig 1A and S3 Table). Besides homopolymeric tracts, strains Ia/CS190/96 and L2b/CS19/08 revealed no mixture of clones, whereas the D/CS637/11, F/CS847/08 and C/TW-3 revealed allelic mixtures (S3 Table). For the serovar D strain, the clones in the population are distinguished by two non-synonymous nucleotide polymorphisms (‘GC’ **↔** ‘AA’) affecting the same codon in the gene CT633/*hemB*, which encodes an enzyme (delta-aminolevulinic acid dehydratase) involved in the heme biosynthesis [77]. A recent study showed that the heme metabolism is important for *C*. *trachomatis* infectivity [78], and, in *Staphylococcus aureus*, it is known that *hemB* mutants display increased ability to persist intracellularly [79]. For the serovar F clinical isolate, the mixture of clones was found to involve a silent mutation in the CT682/*pbpB* gene. This gene codes for a penicillin binding protein that play a role in the final stages of the synthesis of peptidoglycan, which was recently discovered in *Chlamydia* [80]. Corroborating the assumption that this mixture of clones comes from the *in vivo* population, there are *C*. *trachomatis pbpB* sequences in GenBank supporting both nucleotide variants. Finally, concerning the ocular prototype strain C/TW-3, the initial population contained a mixture of CT135-positive and CT135-negative clones, where the minor frequent clones carry one inactivating indel in the gene CT135 (S3 Table). Globally, allelic mixtures were sustained by depth of coverage ranging from ~180x to ~1100x (S1 Table).

### Phase variation may underlie functionality of *C*. *trachomatis* cytotoxin

The *C*. *trachomatis* cytotoxin is a putative effector protein believed to act on the rapid disassembly of cytoskeleton actin filaments during the bacterial internalization process and to cause a cytopathic effect in host cells [81–84]. Contrarily to epithelial-genital strains, LGV and oculotropic strains do not encode a functional cytotoxin [81, 85]. Intriguingly, we found clones in the initial populations of all epithelial-genital isolates displaying a potentially disrupted cytotoxin due to an inactivating variable ‘G’ count in a poly(G) tract at the 5’-end of CT166 (gene positions 29 to 37). In fact, although the predicted functional cytotoxin (“ON”) harbors a string of a 9 Gs, the majority of the sequence reads generated for three epithelial-genital isolates (>83% for D/CS637/11 and F/CS847/08, and >66% for Ia/CS190/96) carry a 10 ‘G’ homopolymeric tract that causes frameshift (“OFF”) (Fig 1C). This is supported by a high depth of coverage from 130x to 470x, and it is known that homopolymer-associated errors are highly reduced when using the Illumina technology [86, 87], which has been used, for instance, for resequencing the variable tracts of contingency loci in *Campylobacter jejuni* [88]. We believe that this variability may underlie the regulation *in vivo* of the cytotoxin functionality through an ON/OFF mechanism of phase variation. In fact, we performed a BLAST search and found out that ‘G’ counts other than nine are present in several recently released culture-independent *C*. *trachomatis* genomes [22].

### *In vitro* passage of *C*. *trachomatis* results in a tropism-specific loss of the virulence gene CT135

**Fig 2:**
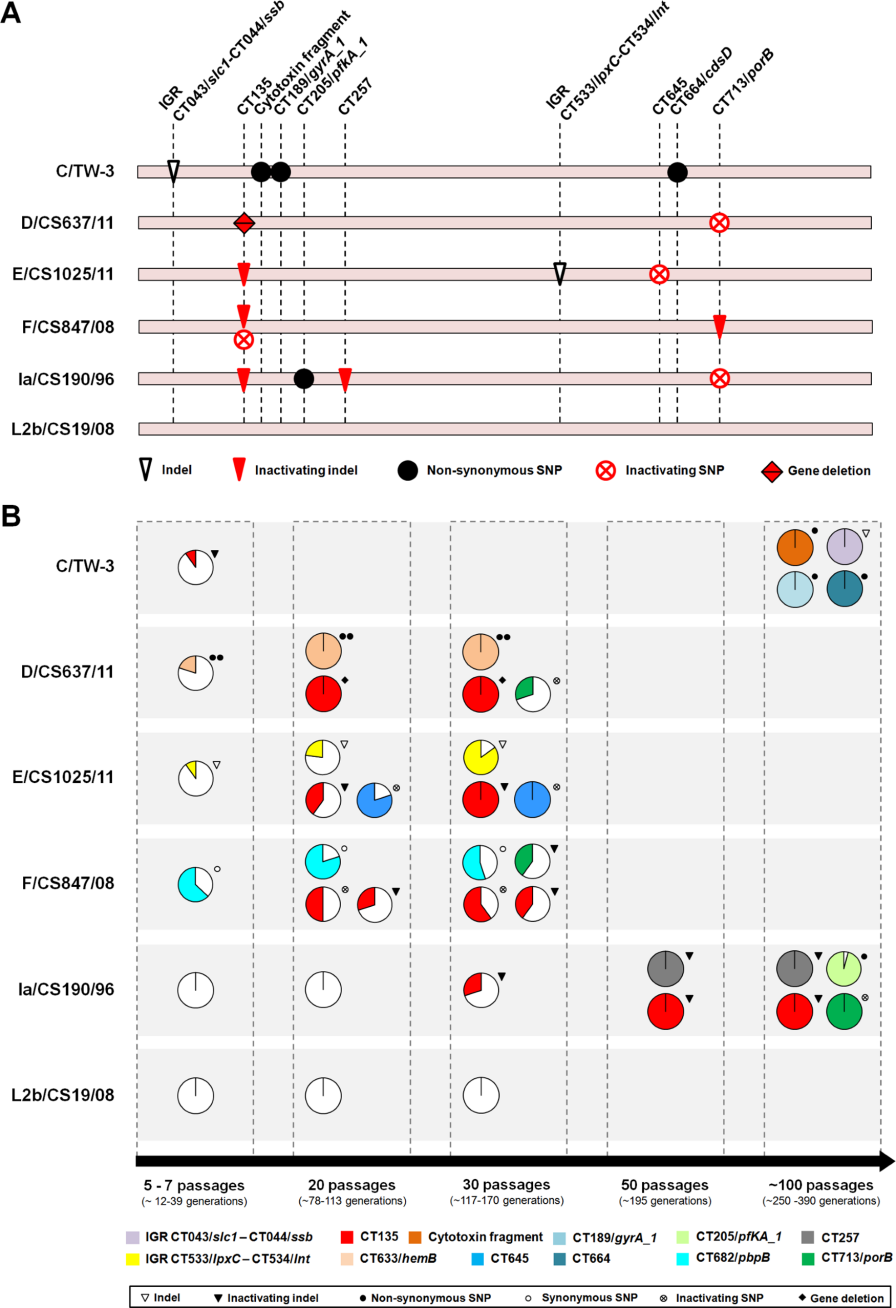
Mutational scenario throughout experimental evolution. Panel A. Chromosomal location of the genomic alterations observed during the *in vitro* passaging. The chromosomal position of each mutation (scale adjusted and given by the locus name) and the type of mutation event (inactivating events represented in red) are shown for each strain (see also S3 Table for details). Inactivating SNPs or indels refer to events leading to protein truncation (regardless the length of the resulting protein). For the strain D/CS637/11, the CT135 inactivating event involved the entire gene deletion between direct repeats (Fig 3). **Panel B**. Dynamics of the emergence and spread of mutations and their frequency in the evolving bacterial populations. For each time-point (passages 5–7, 10, 20, 30, 50 and 100), circular graphs show the frequency of the mutations in the bacterial population, where each color represents a different mutated locus. The number of bacterial generations was estimated taking into account the minimum and maximum values of the mean doubling time of the strains analyzed at each time-point, and assuming a conservative approach by considering 15 hours of exponential phase *per* bacterial life-cycle (i.e, *per* passage). Loci designations are based on genome annotation of the D/UW3 strain (GenBank accession number NC_000117).

**Fig 3:**
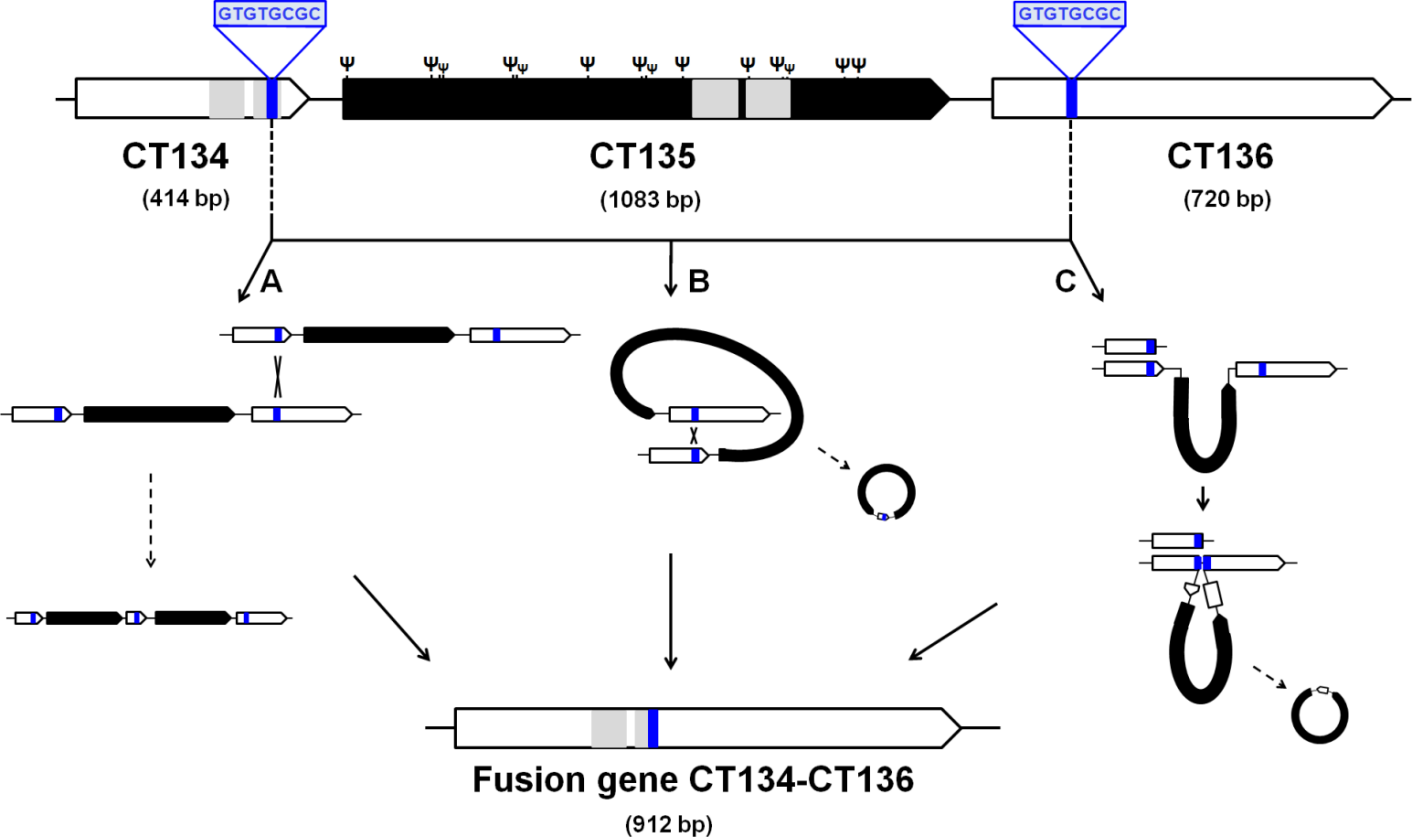
Schematic representation of the CT135 deletion in the serovar D strain. The inactivating event of CT135 involved the complete gene deletion between direct repeats (in blue) and the putative formation of a fusion gene enrolling the two flanking genes (CT134 and CT136). The underlying mechanism likely relied in one of three major pathways: A—intermolecular crossing over between direct repeats followed by recombination (yielding both a tandem duplication and a deletion); B—looping out in between direct repeats followed by recombination; and C—DNA polymerase slippage during DNA replication [89–91]. The figure also shows the position of all CT135 frameshift mutations (labeled by Ψ) reported here and elsewhere [46, 48, 131, 149, 150, 164], demonstrating that the strains evolved towards CT135 inactivation regardless the “genetic pathway” that drove that inactivation The bilobal hydrophobic domains that putatively enable the insertion of the CT134 and CT135 proteins into the inclusion membrane [33] are highlighted in grey.

We evaluated the emergence and spread of adaptive mutations throughout *C*. *trachomatis* experimental evolution (Fig 2). No emergent mutations were detected for the LGV-proctitis strain (L2b/CS19/08) at any time-point. In contrast, we observed inactivating mutations in the virulence gene CT135 for all the epithelial-genital strains, which rapidly rose in frequency. We estimated that, in each 10 *in vitro* passages of epithelial-genital strains, a mean of 23.1% (SD ±11.9) of the emergent clones will carry inactivating mutations in this virulence gene. An extreme example stands for D/CS637/11, where CT135-negative clones reached a frequency of 100% at passage 20. For this strain, the inactivating mutation consisted of a 1452-bp deletion between direct repeats leading to the putative formation of a fusion gene involving the flanking genes CT134 and CT136 (Fig 3). Three major alternative mechanisms may have mediated this event: *i)* intermolecular crossing over between DNA direct repeats; *ii)* intramolecular pairing of repeats by looping out followed by homologous recombination; and *iii)* DNA polymerase slippage during DNA replication [89–91]. Of note, deletions between direct repeats have been proposed to have played a role in the genome reductive evolution of *Chlamydia* bacteria [85, 92, 93], namely in the cytotoxin loss in the ocular *C*. *trachomatis* serovars A, Ba and C [85]. In support of the expression of the fusion protein CT134-CT136, we observed the existence of transcripts (in RNA-seq) compatible with this novel genomic structure. However, immunoblotting attempts to detect the protein (polyclonal antibody targeting CT136) in culture lysates obtained at mid-cycle were unsuccessful (data not shown).

In a later point of the evolution experiment, a parallel scenario of gene inactivation occurred for CT713/*porB* gene for three out of four epithelial-genital strains (Fig 2 and S3 Table). Since this parallelism provides a strong signal of metabolic adaptation of *C*. *trachomatis* to the culture conditions, we will discuss it in detail in the next section. Some other adaptive mutations were fixed in the evolving populations, although no parallelism across strains was observed for the targeted genes. For the serovar E strain, we detected the emergence and evolution to predominance of clones carrying one inactivating SNP in the start codon of CT645, which encodes a predicted integral membrane protein with unknown function (YGGT family) [77] that shows homology to a protein (YlmG) potentially involved in cell division [94, 95]. Also, for the Ia/CS190/96 strain, two new adaptive mutations arose: an inactivating indel for CT257 and one non-synonymous SNP for CT205/*pfkA_1*, both reaching about 100% frequency. CT257 codes for a CBS domain-containing protein [96] that was suggested to display tropism for eukaryotic lipid-droplets [97] whereas CT205/*pfkA_1* encodes a key enzyme (diphosphate-fructose-6-phosphate 1-phosphotransferase) involved in the carbohydrate metabolism [77].

Regarding the ocular strain C/TW-3, we verified that the minor frequent CT135-negative clones present in the initial population evolved to extinction, reflecting a completely opposite scenario to the one observed for the epithelial-genital strains. Additionally, we observed the further fixation of four adaptive mutations reaching 100% frequency at passage 100 (Fig 2 and S3 Table). These mutations targeted the following loci: the intergenic region (IGR) upstream from the gene CT043/*slc1* (a 1-bp deletion in a poly(A) tract affecting the transcript sequence), the genes CT189/*gyrA_1* and CT664/*cdsD*, and a putative ORF (CTW3_00885) [72] encoding a fragment of the ancestral *Chlamydia* cytotoxin. Of note, two of these mutations might have likely interfered with T3S-mediated bacterial functions, since the gene CT043/*slc1* encodes a chaperone (Slc1) of several virulence-associated T3S effectors [98, 99], whereas the gene CT664 encodes a forkhead associated (FHA) domains-containing protein (CdsD/YscD) predicted to form the inner membrane ring of T3S apparatus [100, 101].

Globally, the observed mutations correspond to an empirical substitution rate (we used a conservative approach by considering minimal exponential phases) ranging from 9.86 x 10^−9^ to 1.89 x 10^−8^ mutations *per* base pair *per* generation (or 0.0103–0.0197 mutations *per* genome *per* generation), which fits data from adaptive evolution studies in bacteria, where evolving populations are expected to fix no more than ~2.5 mutations *per* 100 generations [4, 6, 102]. Of note, no mutations were observed in plasmid sequences, and the number of plasmids *per* chromosome did not significantly change throughout laboratory propagation (S1 Table).

### Inactivating mutations in the gene CT713/*porB* may reflect metabolic adaptation to the available carbon source

Three out of the four epithelial-genital strains acquired putative inactivating mutations in CT713/*porB* that increased in frequency (Fig 2 and S3 Table). CT713/*porB* gene codes for a porin that specifically enables the uptake of dicarboxylates into the tricarboxylic acid (TCA) cycle [103]. This cycle is incomplete in *C*. *trachomatis*, and requires either exogenous glutamate (transported by the major outer membrane protein—MOMP) or 2-oxoglutarate (transported by PorB) from the host cell [77, 103, 104]. Hence, as we supplemented the chamydial cultures with glutamate, the 2-oxoglutarate uptake pathway might become expendable, leading to both the inactivation of the porin-encoding gene CT713/*porB* and exclusive maintenance of the TCA feeding through the glutamate pathway. This hypothesis would rely on a typical scenario of metabolic adaptation to the available carbon source, and fits well with the postulation that the reduction of importance of the TCA cycle metabolic pathway throughout the adaptation of *Chlamydiae* to an intracellular lifestyle led to the pseudogenization of TCA cycle-related genes [105].

### The growth rate of laboratory evolving populations increased relative to the ancestral populations leading to shorter life-cycles

**Fig 4:**
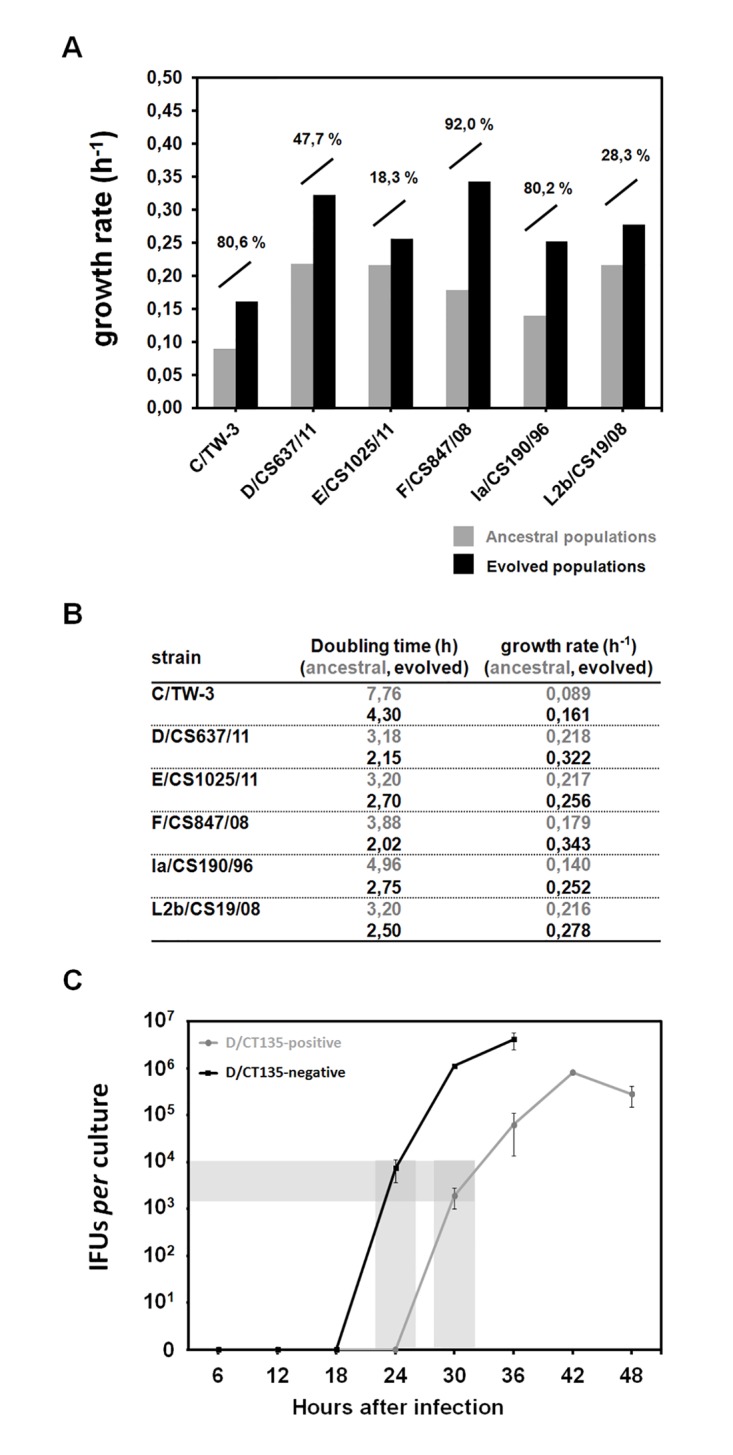
Impact of *in vitro* passaging on the *C*. *trachomatis* growth kinetics. Panels A and B. Comparison of the growth rates and doubling times between ancestral (grey) and evolved populations (black). The percentage values above the bars correspond to the growth rate increment of the evolved population relatively to the ancestral. Panel C. Comparison of the one-step growth curve between D/CS637/11 CT135-positive and CT135-negative strains. Cells grown in the same conditions were infected at a MOI of 1, and cell scrapings were collected over time after infection for analysis of inclusion-forming units (IFUs). The black line represents the evolved CT135-negative D/CS637/11 strain, whereas the grey line represents the ancestor CT135-positive strain. The shaded area indicates the time points chosen for RNA-seq differential expression comparative analyses.

In *Chlamydia*, fitness estimates essentially rely on evaluating the growth rates or IFU yield rather than on capturing the phenotype by which the adaptive advantage operates, as knock-out assays are not optimized yet for such purpose. Thus, we searched for changes in the fitness of the evolving populations by comparing the growth rates of the strains at the beginning and at the end of the study, a methodology largely applied in bacterial experimental evolution studies [3]. Globally, the growth rate of all evolved populations relative to their ancestors increased from 18,3% (for E/CS1025/11) to 92,0% (for F/CS847811) (Fig 4A and 4B), reflecting the progressively shortening of the bacterial life-cycles observed through phase-contrast microscopy. In addition, we complemented this data by constructing a one step-growth curve (i.e., enumeration of IFUs over time) to assess the length of the life-cycle before and after the CT135 loss by the serovar D strain (Fig 4C). This assay revealed a shorter developmental cycle for the propagated strain, which was also sustained by the observation of a similar fold change in the genome copy number between 4h pi to 30h for the D/CT135-positive strain and 4h pi to 24h for the D/CT135-negative strain (data not shown).

### The genomic inactivation of CT135 in the epithelial-genital strains is accompanied by a quick degradation of the mRNA

**Fig 5:**
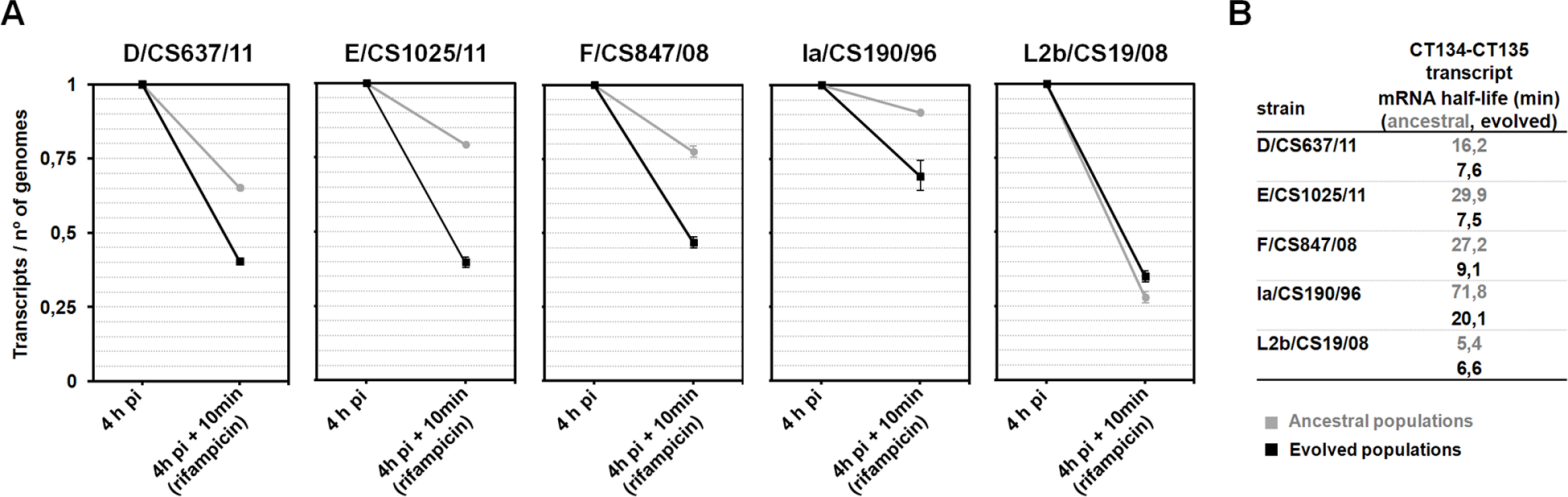
CT135 mRNA decay analysis. Panel A shows the comparison of the relative amount of transcripts at 4 h post-infection (pi) and after 10 min of transcriptional blockage with rifampicin (10 μg/ml) between the ancestral (grey) and the evolved populations (black). The assay was performed for all strains with emergent CT135-negative clones (i.e., all epithelial-genital isolates) and for the strain L2b/CS19/08 (control). The number of transcripts was quantified by independent RT-qPCR targeting the two genes of the operon CT134-CT135 (see methods for details), except for serovar D strain as the evolved population lacks CT135. Data was normalized against the number of *C*. *trachomatis* genomes quantified on the corresponding DNA samples. In order to facilitate the comparative analysis, the normalized value before rifampicin treatment (4h pi) was arbitrarily set to 1. Panel B shows the mRNA half-life times calculated based on the fit of an exponential decay between the quantified values at 4h pi and the values calculated 10 minutes after the transcriptional arrest.

The mechanisms for controlling the mRNA processing and decay play an important role in the continuous adjustment of the levels of gene expression according to the protein needs, with bacterial mRNA half-life times ranging from seconds to hours [62, 106]. We evaluated whether the molecular stability of CT135 transcripts was affected after protein truncation. We observed that the mRNA half-life time decreased for the epithelial-genital isolates (Fig 5), whereas it remained unaltered for the LGV clinical isolate. These results are concordant with an adaptive scenario where both the genomic inactivation and mRNA processing mechanisms concomitantly act to increment the competitive fitness of the CT135-negative clones. Curiously, when looking at the mRNA half-life time of the ancestral strains, we observed that the CT135-transcript is more labile for the LGV strain.

### Genomic deletion involving CT135 affects the expression of multiple virulence-associated genes

**Fig 6:**
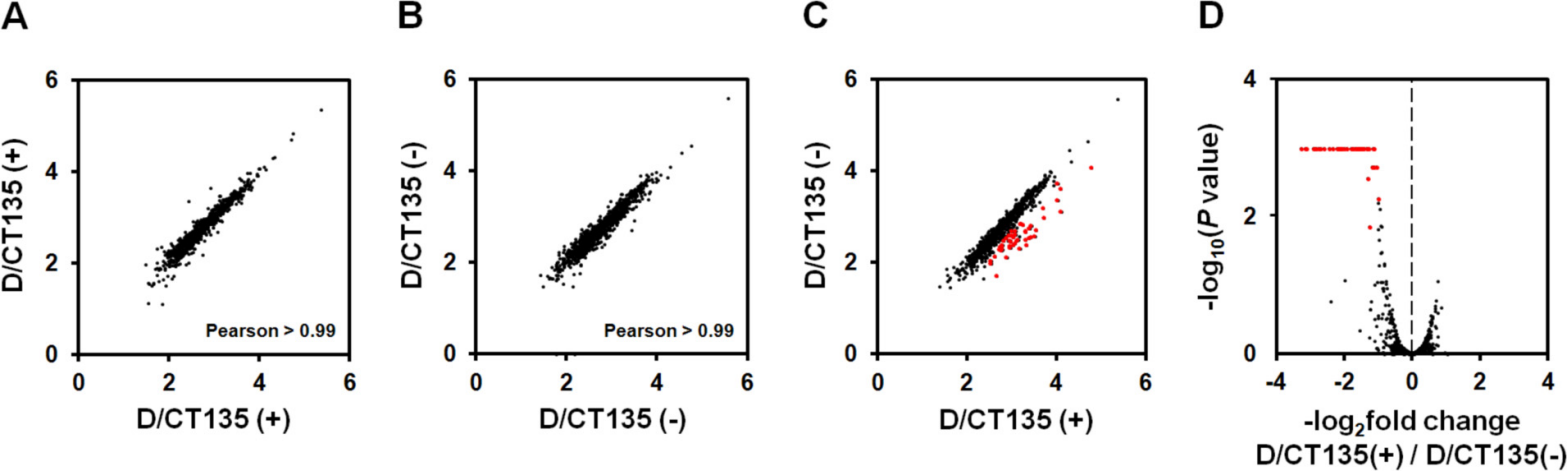
Comparative analysis of global gene expression (RNA-seq) between D/CT135-positive and D/CT135-negative populations. Panels A-B. Comparison of gene expression between biological replicates for the D/CT135-positive (A) and D/CT135-negative (B) populations. Pearson correlation coefficients are shown. Panel C. Comparison of gene expression between the D/CT135-negative and D/CT135-positive populations. The red points mark genes and the non-coding RNA for which the fold change of expression exceeds two-fold and the FDR-corrected *P*-values were below 0.05. For panels A to C, axes are log_10_-transformed normalized expression levels (FPKM). Panel D. Volcano plot of –log_2_ fold change (D/CT135-positive *versus* D/CT135-negative) *versus* –log_10_ adjusted *P*-values. In order to better fit the scale to data, corrected *P*-values ≤10^−3^ were set as 10^−3^. Points in red indicate genes and the non-coding RNA for which the fold change of expression exceeds two-fold and the FDR-corrected *P*-values were below 0.05.

The mechanism by which CT135 promotes *C*. *trachomatis* virulence is unknown. Taking into account the rapid and parallel loss of the CT135 in all epithelial-genital strains, we wonder if the *in vitro* inactivation of CT135 is beneficial likely because it reduces the expression of several genes, and hence the associated energetic costs of unused functions. We performed RNA-seq analyses to compare the global gene expression of the serovar D CT135-positive and CT135-negative strains. Here, we are assuming that potential changes in gene expression are essentially due to the CT135 loss, but the deletion of 1452-bp also partially involved the flanking genes CT134 (encodes a putative Inc protein) and CT136 (encodes a Lysophospholipase esterase) (Fig 3), so a synergistic effect cannot be ruled out. This particularly stands for CT134, as it is predicted to belong to the same operon as CT135 [46]. By using a false discovery rate cutoff of 0.05 and a fold-change cutoff of >2.0, we identified 48 significantly differentially expressed genes and one non-coding RNA, all being down-regulated in the CT135-negative strain (Table 1 and Fig 6). To confirm these results, we used RT-qPCR to evaluate the expression of a set of the highlighted genes and found a good level of correlation (slope 1.01, Pearson correlation 0.958, N = 7) (S2 Fig). Remarkably, the pool of genes that are down-regulated after the occurrence of the deletion event involves multiple virulence-associated genes (some of them belonging to the same operon) (Table 1). We highlight genes coding for: *i*) the most prominent adhesins (e.g., CT443/OmcB) [107, 108] and T3S effectors (e.g., CT456/Tarp, CT694 and CT875/TepP) known to play a role in the *C*. *trachomatis* host-cell invasion process [109–112]; *ii*) multiple T3S-related proteins, such as substrates (e.g., CT082, CT619-20 and CT847-9) [38, 99, 113–115], a chaperone (CT576/Scc2) [116] and components of the translocation pore (e.g., CT578/CopB and CT579/CopD) [117]; *iii*) genes encoding proteins putatively related to the chlamydial protease/proteasome-like activity factor (CPAF), either potential substrates (e.g., CT005, CT288, CT443/OmcB, CT456/Tarp and CT694-5) [118–120] or other virulence proteases acting in the same pathways in the subversion of host-cellular functions (e.g., CT441/Tsp and CT868/ChlaDub1) [121–124]; and *iv*) virulence-associated proteins regulated by the plasmid-encoded Pgp4 (e.g., CT049-CT051, CT142-CT144 and CT798/GlgA) [51]. Regarding the latter set of genes, we looked at the differential expression of their regulator-encoded gene (ORF6/*pgp4*) and observed a slight decrease of expression (~1.4-fold) in the CT135-negative strain. Thus, we hypothesize that the down-regulation of those genes might have been mediated by *pgp4* underexpression. Of note, the down-regulated genes include all members (CT619, CT620, CT621, CT711, and CT712) except CT621 of a family of chlamydial T3S substrates characterized by a domain of unknown function (DUF582 proteins) that are believed to target nuclear cell functions [115, 125]. The highly down-regulated non-coding small RNA (sRNA) (located between CT080 and CT082, and previously designated ctrR0332 in the L2b/UCH-1 strain) (Table 1) was previously found to be overrepresented in EBs, which is consistent with the profile found for the majority of the affected transcripts [126]. Noteworthy, we observed only one differentially expressed gene (CT377/*ltuA*) in the control experiment with the Ia/CS190/96 strain. This result suggests that the short-term laboratory propagation of *C*. *trachomatis* does not substantially change gene expression in absence of genomic alterations.

**Table 1:** Genes and a non-coding RNA found to be down-regulated in the serovar D CT135-negative strain.

locus ^a^ ^,^ ^b^	log_2_[fold change D/CT135(+) / D/CT135(-)] ^c^	Putative role / experimental evidence
**sRNA** ^d^	-3,274	Abundant non-coding RNAs differentially expressed in EBs and RBs [126].
**CT082** ^e^	-3,153	T3S substrate [99, 165]. Role in host cell invasion and infectivity? [126].
**CT814.1**	-3,139	Putative Inc [33]. Role in host cell invasion and infectivity? [126].
**CT814**	-3,118	Role in host cell invasion and infectivity? [126].
**CT635**	-2,913	---
**CT579/*copD*** ^f^	-2,880	T3S translocation pore component (CopD) [117]. Role in host cell invasion and infectivity? [126].
**CT578/*copB*** ^f^	-2,825	T3S translocation pore component (CopB) [117]. Role in host cell invasion and infectivity? [126].
**CT576/*scc2*** ^f^	-2,762	T3S chaperone Scc2 [116]. Role in host cell invasion and infectivity? [126].
**CT577** ^f^	-2,693	Role in host cell invasion and infectivity? [126].
**CT005**	-2,591	Putative Inc [166]. Putative CPAF substrate [119]. Role in host cell invasion and infectivity? [126].
**CT080/*ltuB***	-2,438	Late transcription unit B protein [167]. Role in host cell invasion and infectivity? [126].
**CT444/*omcA*** ^f^	-2,348	Cysteine-rich outer membrane protein. Role in host cell invasion and infectivity? [126].
**CT443/*omcB*** ^f^	-2,214	Cysteine-rich outer membrane protein [107]. Adhesin [107, 108]. Role in host cell invasion and infectivity? [126]. Putative CPAF substrate [118].
**CT848** ^f^	-2,180	Putative T3S substrate [38]. Role in infectivity in *C*. *muridarum*? [127].
**CT456/*tarp***	-2,162	Translocated actin-recruiting phosphoprotein (TARP) / early T3S effector prepackaged into EBs [110]. Involved in host cell invasion [112]. Putative CPAF substrate [119].
**CT875/*tepP***	-2,152	Translocated early phosphoprotein (TepP) / early T3S effector involved in host cell invasion [109]. Role in host cell invasion and infectivity? [126].
**CT694** ^f^	-2,140	Early T3S effector (prepackaged into EBs) involved in host cell invasion [111]. Putative CPAF substrate [119].
(CT622)	-2,120	Effector [168]. Putative plasmid-related virulence protein [51]. Role in host cell invasion and infectivity? [126].
**CT847** ^f^	-2,108	T3S effector putatively involved in the modulation of the cell cycle [113].
**CT181**	-2,093	Role in host cell invasion and infectivity? [126].
**CT046/*hctB***	-2,090	Histone-like protein 2. Mediate the DNA condensation that is characteristic of EBs [169].
**CT849** ^f^	-2,017	T3S substrate [114]. Implicated in *C*. *muridarum* adherence to host cells [16].
**CT620**	-1,925	T3S effector (DUF582 family) believed to target nuclear functions [115, 125]
**CT392/*yprS***	-1,807	—
**CT619**	-1,738	T3S effector (DUF582 family) believed to target nuclear functions [115, 125].
(CT798/*glgA*)	-1,736	Glycogen synthase. Plasmid-related virulence protein [51]. Effector [170].
(CT051) ^f^	-1,670	Plasmid-related virulence protein [51]. Pmp-like protein [166, 171].
**CT441/*Tsp***	-1,651	Effector. Tail-specific protease. Subversion of host cell functions by preventing the host NF-κB activation [121, 122, 124].
**CT868/*ChlaDub1***	-1,648	Effector with deubiquitinating and deneddylating activity (protease) [172]. Subversion of host cell functions by suppressing the NF-κB activation [123].
**CT356/*yyaL***	-1,631	Thioredoxin domain-containing protein [96]. Role in host cell invasion and infectivity? [126].
**CT695** ^f^	-1,623	Early putative T3S effector prepackaged into EBs [111]. Putative CPAF substrate [120].
**CT288**	-1,619	Putative Inc [173]. T3S substrate [38]. Role in host cell invasion and infectivity? [126]. Putative CPAF substrate [119].
(CT565)	-1,572	Putative Inc [33]. Putative plasmid-related virulence protein [174].
**CT546**	-1,542	Predicted outer membrane protein [175].
**CT712** ^f^	-1,525	Putative T3S effector (DUF582 family) believed to target nuclear functions [115, 125]. Putatively packaged into EBs [111].
**CT365**	-1,459	Putative Inc [33]. T3S substrate [109]. Role in host cell invasion and infectivity? [126].
(CT702)	-1,441	Putative plasmid-related virulence protein [51, 174].
**CT214**	-1,435	Putative Inc [165]. Role in host cell invasion and infectivity? [126].
**CT711** ^f^	-1,334	Putative T3S effector (DUF582 family) believed to target nuclear functions [115, 125].
(CT049) ^f^	-1,325	Pmp-like protein secreted into the inclusion lumen [166, 171]. Putative plasmid-related virulence protein [51].
**CT837**	-1,300	---
**CT052/*hemN_1***	-1,275	Coproporphyrinogen III oxidase [77].
(CT144) ^f^ ^,^ ^g^	-1,244	T3S substrate [114]. Putative plasmid-related virulence protein [51].
(CT142) ^f^ ^,^ ^g^	-1,180	T3S effector [114]. Putative plasmid-related virulence protein [51].
**CT372/*oprB***	-1,155	Outer membrane protein with a carbohydrate-selective porin (OprB) [176].
(CT050) ^f^	-1,125	Pmp-like protein secreted into the inclusion lumen [166, 171]. Putative plasmid-related virulence protein [51].
**CT564/*yysT***	-1,114	T3SS Integral membrane structural protein (Yop proteins translocation protein T) [177].
**CT735/*dagA_2***	-1,037	Na(+)-linked D-alanine glycine permease [77].
**CT659**	-1,010	Predicted RNA binding protein [67]. Role in host cell invasion and infectivity? [126].

^a^ Loci nomenclature and numbering refer essentially to the annotated genome from the D/UW3 strain (GenBank accession number NC_000117).

^b^ Loci in bold are potentially down-regulated on behalf of the CT135 loss. Non-bold loci (also in parentheses) were previously found to be transcriptionally regulated by the plasmid (namely by the plasmid-encoded protein Pgp4) [51, 174]. In the present study, the down-regulation of this latter set of genes may be associated with Pgp4 as it was found to display lower expression levels in the D/CS637/11 CT135-negative population.

^c^Adjusted P-values < 0.05 and a fold-change cutoff > 2. Genes are ordered according the magnitude of differential expression.

^d^ Refers to the previously identified sRNA ctrR0332 [126] that actually comprehends two sRNAs (likely processed from a larger transcript). In the D/UW-3 annotation, it is located inside a non-existing but previously annotated ORF (CT081). Both sRNA were similarly down-regulated.

^e^ CT082 was suggested to be up-regulated in the *pgp4* knockout mutants [51]. As in the present study it was found to be strongly down-regulated even with a slight *pgp4* expression decrease, we assumed this result as a consequence of CT135 loss.

^f^ These genes are likely expressed in the same transcriptional unit as other down-regulated contiguous genes [also marked].

^g^ Although it is believed that the CT143 gene is coordinately expressed with the flanking genes CT142 and CT144 [114], the obtained differential expression value was slightly below the cutoff.

## Discussion

It is known that pathogens adapt to laboratory culture conditions, whereby *in vitro*-maintained strains may no longer reflect the circulating isolates. On behalf of this, the use of laboratory-passaged strains has been frequently questioned in *Chlamydia* research [17, 20–24]. Although the adaptive process behind the *in vivo* to *in vitro* transition is not understood, cumulative data have pointed out that a deep characterization of this process is mandatory, since: *i*) mixed-clone populations have been found in multiple culture stocks from both *C*. *trachomatis* and *C*. *muridarum* strains, with clones displaying distinct *in vivo* virulence [23, 46, 127, 128]; *ii*) prototype strains have been suggested to display distinct phenotypic presentations when comparing with recent clinical isolates, such as distinct growth-rates and/or yielded progeny [21, 129] and *in vivo* infectivity capacities [20]; *iii*) *in vitro* passage selected for *C*. *muridarum* displaying severely attenuated *in vivo* pathogenicity [16]; *iv*) clones with frameshift mutations in the *C*. *trachomatis* virulence gene CT135 (or in the *C*. *muridarum* homolog TC_0412) have been detected in several cultured populations [15, 17, 23, 46, 48, 127, 128, 130, 131]; and *v*) the use of cell-culture propagation in *Chlamydia* research will expectedly be boosted due to the recent development of transformation and gene knockout experiments in *Chlamydia* [48–52, 132–135].

In the present study, we specifically aimed to assess the genomic and transcriptomic dynamics of *C*. *trachomatis* after its introduction in culture. As the experimental design of the present study mirrored the typical culture techniques, i.e., assisted bacterial entry and exit (by centrifugation and sonication, respectively), which discards the selective pressure underlying these steps, no discussion will be performed about evolutionary adaptation targeting specifically the bacterial attachment and host-cell lysis. This would require a study with different design and goals [16]. We observed a dynamics of mutation fixation for this obligate intracellular bacterium that clearly fits the typical scenario of adaptive evolution seen in similar experiments in extracellular bacteria [1, 6]. The most frequently observed events were inactivating mutations followed by non-synonymous mutations (none of the emergent mutations in ORFs were synonymous), and the mutant clones progressively rose in frequency in the population, clearly supporting that mutations were beneficial (Fig 2). In general, our results show an adaptive scenario underlying the *in vivo* to *in vitro* transition for epithelial-genital strains that seems to be essentially marked by two major stages within different time-frames: *i*) a rapid and parallel evolutionary loss of the virulence gene CT135; and *ii*) a later inactivation of the porin PorB (encoded by the gene CT713/*porB*), which may be a result of a metabolic adaptation to the carbon source (glutamate instead of 2-oxoglutarate). Regarding the latter, it is intriguing why not all strains were affected, which we speculate to be a matter of time-scale. Future experiments involving the switch between glutamate and 2-oxoglutarate coupled with the analyses of the population dynamics will certainly dissect this hypothesis. These results provide an explanation to the enigmatic emergence of both CT135-null [15, 46, 48, 131] and *porB*-null mutants [136] in *in vitro* populations. Notably, CT135-negative clones rapidly outcompeted CT135-positive clones, suggesting a strong selective force against CT135 functionality. Although we cannot exclude that inter-clone recombination may have also contributed to this notable frequency increase, values of this magnitude were only previously observed under strong selective pressures with antichlamydial compounds [137].

CT135 encodes a putative Inc [33] that contains a T3S signal recognized by heterologous bacteria [32], but whose function is unknown. We found that CT135 may be a direct or indirect virulence regulator, as its genetic loss together with the partial deletion of the two flanking genes implicated a down-regulation of multiple genes, where more than 30 have been previously associated with *C*. *trachomatis* virulence properties (Table 1). We hypothesize that the down-regulation of virulence genes constitutes the mechanism behind the observation that CT135-null mutants showed attenuated *in vivo* infections in mice [46, 47]. In support of this, an interesting recent study from Ramsey’s group [17] showed that when mice were inoculated with *C*. *muridarum* population displaying a mixture of positive and negative clones of TC_0412 (CT135 homolog), the former shift to predominance. Although it is believed that CT135 is an Inc protein, its location in the inclusion membrane was never demonstrated by immunofluorescence microscopy. Yet, CT135 is most expressed at the beginning of *C*. *trachomatis* life-cycle [32, 63], coincident with the formation of the inclusion membrane, and belongs to the same operon and displays the same expression levels and profile as CT134, which also encodes a putative Inc protein [32, 33, 46]. As the C-terminal region of CT134 was also lost in the deletion event, we could also speculate the existence of a cooperative action involving CT134 and CT135, where the CT135 effect would be boosted by the upstream protein.

Almost all affected virulence factors seem to be coordinately expressed at late or immediately early stages of *C*. *trachomatis* life-cycle [32, 62, 66, 67, 114, 126], suggesting their packaging into EBs for subsequent rounds of infection. Curiously, some of them were previously shown to be transcriptionally regulated by the well-described EUO (e.g., CT443/*omcB*, CT080/*ltuB*, CT441/*Tsp* and CT576/*scc2*), which similarly to CT135 is also an early protein [138, 139], or by the plasmid Pgp4 (e.g., CT049-CT051, CT142-CT144, CT622 and CT798/*glgA*) [51]. Whereas CT446/*euo* revealed no changes in expression, the slight lower expression of *pgp4* may justify the detected down-regulation of Pgp4-targets. This latter observation leaves open the possibility that the attenuation of *C*. *trachomatis* virulence *in vitro* may occur by more than one mechanism (such as genomic inactivation of CT135 and/or down-regulation of the virulence regulator Pgp4), which potentially take place in a cumulative or differential fashion depending on the strain. Future transcriptional analyses of other *in vitro* passaged strains, namely CT135-unaltered strains and strain with an extent passaging time-scale, will certainly be important to confirm this hypothesis.

The most down-regulated locus (about 10-fold) was the previously described sRNA ctrR0332 [126]. Although increasing data have shown that the expression of sRNAs may be determinant for virulence in both extracellular [140] and intracellular bacterial pathogens [141] and, in *C*. *trachomatis*, one sRNA (lhtA) was found to control the timing of RB to EB transition [142, 143], the specific role of the sRNA ctrR0332 on *C*. *trachomatis* virulence cannot be determined yet. However, it is tempting to hypothesize that it may regulate CT135 (for instance, by controlling its mRNA stability), whose loss would render the sRNA dispensability. Collectively, all these data indicate that the control of *C*. *trachomatis* virulence at the stage where EBs are loaded with virulence determinants is likely multi-factorial.

The scenario of the rapid loss of CT135 in the transition from *in vivo* to *in vitro* raises relevant issues concerning studies where intensive laboratory propagation is necessary, such as mutagenesis, transformation and/or drug testing. In fact, these may enhance the selection of CT135-negative mutants, where a single inactivating event may have a downstream strong impact on the virulence and pathogenicity of the strains, introducing bias when interpreting data on the course of their use. For example, the loss of virulence after *in vitro* propagation is well-documented for other important human pathogens, such as *Mycobacterium tuberculosis* [10], *Staphylococcus aureus* [12], *Coxiella burnetii* [11] and *Campylobacter jejuni* [144]. Therefore, it seems important that researchers performing studies with laboratory-propagated *C*. *trachomatis* epithelial-genital strains confirm the integrity of CT135 in selected clones. This issue is also raised when working with *C*. *muridarum*, as the interpretation of the results from recent studies searching for targets of antibiotic resistance [130] or evaluating pathogenicity *in vivo* [16] was complicated by the presence of frameshift mutation in CT135-homolog. On the other hand, this issue can be overcome if complementation assays are applied to study the genetic linkage between a mutant allele and the expected phenotype.

One intriguing result was the observation that CT135 inactivation occurred for all epithelial-genital strains (regardless of both their genetic backbone and the clinical prevalence of their representative serovars), but not for strains with different tissue tropism (LGV and ocular strains). Considering that *C*. *trachomatis* is likely undergoing a directional evolution towards niche-specific adaptation to each infected tissue—ocular conjunctivae, genital mucosa and lymph nodes [58, 74, 145, 146], one might speculate that CT135 may be a direct or indirect determinant of tissue tropism, as suggested for other *C*. *trachomatis* loci (e.g., the cytotoxin gene and the tryptophan operon) [82, 147, 148]. Several lines of evidence sustain that the LGV strains evolve to retain the CT135 activity *in vitro*: *i*) the passaging of the LGV-proctitis associated clinical isolate do not lead to the CT135 disruption (Fig 2); *ii*) CT135-null mutants did not arise even when the historical LGV prototype strain (L2/434/Bu) was continuously propagated in HeLa cells during over one year [15]; and *iii*) no CT135 polymorphisms were found within the population of any prototype strain from different LGV serovars (L1, L2 and L3) [46]. Regarding the ocular strain that we have used (C/TW-3), we observed a scenario where a sub-population of CT135-null clones evolved to extinction (Fig 2 and S3 Table), which is enigmatic since the CT135 was already found to be disrupted in several annotated genomes of ocular strains [48, 58, 149, 150]. Considering all these observations, and although the HeLa cell line is one of the most commonly used for culturing *C*. *trachomatis*, we wonder if a similar scenario is obtained by propagating LGV and ocular strains in tropism-related cell-lines, such as monocytic or conjunctival cell lines, respectively. CT135 transcripts have been found to be more labile for the LGV strain (Fig 5), but this does not directly mean that CT135 does not contribute to the ability of *C*. *trachomatis* LGV strains to infect macrophages and disseminate to lymph nodes. In fact, the CT135 protein sequence has five LGV-specific amino acids (data not shown), and was previously found to be among a pool of genes likely involved in phenotypic differences among LGV strains [54].

The growth rate of all laboratory evolving populations increased relative to the ancestral populations (leading to shorter life-cycles), reflecting a gradual Darwinian fitness improvement over time (Fig 4). Although we cannot attribute the life-cycle shortening to the inactivation of CT135 (since the ocular and the L2b strains also improved their fitness, and other mutations arose), it is noteworthy that *in vivo* studies also suggested that CT135 operates in sustaining a more persistent or chronic infections in the female mouse genital model [47]. Other fixed mutations (Fig 2 and S3 Table) have also the potential for altering the bacterial fitness likely by affecting: *i*) the cell division (mutation in CT645); *ii*) the nutrient acquisition/processing (mutations in CT205/*pfkA_1*, CT257 and CT713/*porB*); or *iii*) global regulatory mechanisms, such as DNA supercoiling (mutation in CT189/*gyrA_1*). Finally, despite most gene targets showed no parallelism across strains (at least for the time-scale evaluated), we cannot discard that these mutational events may be related to the CT135 loss [either functionally or genetically (e.g., hitchhiking)].

In the present experimental evolution study, we also found genetic features potentially underlying phase variation mechanisms in *C*. *trachomatis*. Phase variation may be driven by reversible alterations in the genotype that causes either frameshift or mutations in non-coding regions impacting gene expression (Fig 1D), and has been associated with genes involved in host-pathogen interactions [151, 152]. Within *Chlamydiaceae*, to our knowledge, phase variation mediated by homopolymeric tracts was only hypothesized to occur in *C*. *pneumoniae*, affecting members of the paralogous gene families Pmps [153, 154] and Cpn 1054 [155]. Here, we detected variable homopolymeric tracts that have the potential to modulate the functionality of genes encoding proteins likely involved in *C*. *trachomatis* pathogenesis: a poly(G) tract within CT166 (encodes the cytotoxin [81]) and a poly(A) tract in the promoter region of CT533/*lpxC* [encoding a critical enzyme in the biosynthesis of lipid A of lipopolysaccharide (LPS) [76]] (Fig 1). Regarding the cytotoxin, variable ‘G’ counts yielding an ON/OFF protein (also present in genomes obtained directly from clinical swabs) suggest the putative existence of cytotoxin-mediated phenotypic diversity within *in vivo C*. *trachomatis* populations of epithelial-genital strains. Still, we wondered if the frameshift mutation in the poly(G) tract could lead to the production of an alternative shorter protein (still including the UDP-glucose binding and glycosiltransferase domains), since the translation could eventually be reinitiated at an alternative putative start codon at position 93 (as frequently annotated in *C*. *trachomatis* genomes deposited in GenBank). However, RNA-seq analyses were not helpful to elucidate this subject as no transcripts were detected (data not shown), hampering the clarification of the two hypotheses. Besides its known activity as an effector in disassembling the host cytoskeleton upon bacterial invasion [81, 83, 84] and it is putative role in subversion of other cellular functions [82], the chlamydial cytotoxin was proposed to account for the resistance to the anti-bacterial effect of IFN-γ [94, 156–158]. In particular, the *C*. *trachomatis* cytotoxin was hypothesized to target human guanylate binding proteins (hGBPs) that are potentiators of the IFN-γ activity (i.e., depletion of the essential amino acid tryptophan) [159, 160]. Therefore, although epithelial-genital strains are capable of using indole for tryptophan biosynthesis [147], we hypothesize that this putative phase variation mechanism may contribute for the quick adaptation of *C*. *trachomatis* to changes in the tryptophan availability by targeting IFN-γ-inducible hGBPs. Concerning the poly(A) tract upstream from CT533/*lpxC*, we observed an increase in frequency of clones carrying dissimilar base counts (Fig 1A and S3 Table), supporting selective advantage. It affects the highly conserved length of the spacer region between the predicted -35 and -10 ‘TATA’ boxes (Fig 1B and 1D), which might alter the binding of the RNA polymerase to the promoter, and thus, the gene expression. We also found this polymorphism in GenBank annotated genomes. Curiously, in a pioneer study back to 1990s [161], it was proposed that the *Chlamydia* LPS is phase-variable, since LPS phenotypic variation was found within a population in tissue culture. In other bacteria, including intracellular organisms, phase variation mechanisms affecting the LPS biosynthesis are well-documented to be associated with antigenic variation [162]. For instance, it was demonstrated that a phase variation-mediated antigenic shift of LPS modulates the ability of *Francisella tularensis* to grow within macrophages [163]. In this regard, a phase variation mechanism targeting the CT533/*lpxC* in *C*. *trachomatis* may control the biosynthesis of lipid A, and ultimately LPS antigenic variation mechanisms for evading the host immune system. Although it was previously demonstrated that *C*. *trachomatis* fails to generate infectious EBs in the presence of LpxC inhibitors [76], putative alterations in the expression of CT533/*lpxC* mediated by the differential length of the promoter spacing did not seem to hamper the progression of chlamydial infection as the progeny of serovar E strain was not affected. Curiously, we noted that the CT134-CT135 transcript has also a poly(T) located between the TSS and the start codon of CT134 (Fig 1D). Although no assumption can be done on its phenotypic impact, a GenBank search revealed a rather intriguing profile of nine, eight and seven ‘T’ counts for most LGV strains, ocular and epithelial-genital strains, respectively. Globally, although the existence of heterogeneity within homopolymeric tracts in *C*. *trachomatis* populations seem to be more common than previously expected and to potentially modulate important bacterial functions, their role in mediating phase variable phenotypes with impact on pathogenesis still needs experimental confirmation. In this regard, phenotypic assays coupled with genetic reversibility of phase variation will soon be tested.

As concluding remarks, our study focused on the *C*. *trachomatis in vivo* to *in vitro* transition reveals not yet identified putative phase variation mechanisms targeting genes involved in pathogenesis, and also potentially contributes to the clarification of the molecular basis underlying the CT135-mediated virulence. CT135 may be a master player in pre-loading EBs with virulence effectors required for the invasion and subversion of epithelial cells. More, as the length of time in the *in vitro* culture is a predisposing factor that contributes to the loss of this virulence gene in epithelial-genital strains, this may constitute an issue as studies demanding culture propagation might enhance the selection of CT135-negative mutants, originating less virulent strains. Thus, restricting laboratory propagation to minimal passages, evaluating the status of the CT135 genotype and performing complementation of mutants might constitute good premises towards both a better interpretation of phenotypic data and inter-laboratory comparisons.

## Supporting Information

S1 Fig Genome make-up of the studied *C*. *trachomatis* strains and evaluation of putative mosaic structures. (PDF)

S2 Fig Confirmation of the RNA-seq results with RT-qPCR. (PDF)

S1 Table Sequence data from the strains analyzed in this study. (PDF)

S2 Table Oligonucleotide primers used in PCR and qPCR assays. (PDF)

S3 Table Genomic alterations throughout *in vitro* passaging. (PDF)
