# Supplementary material for: Chlamydia trachomatis In Vivo to In Vitro Transition Reveals Mechanisms of Phase Variation and Down-Regulation of Virulence Factors
Source: PLoS One. 2015 Jul 24;10(7):e0133420. doi: 10.1371/journal.pone.0133420 (PMC4514472; doi:10.1371/journal.pone.0133420)
Supplement: S1 Fig — (PDF) [file pone.0133420.s001.pdf]

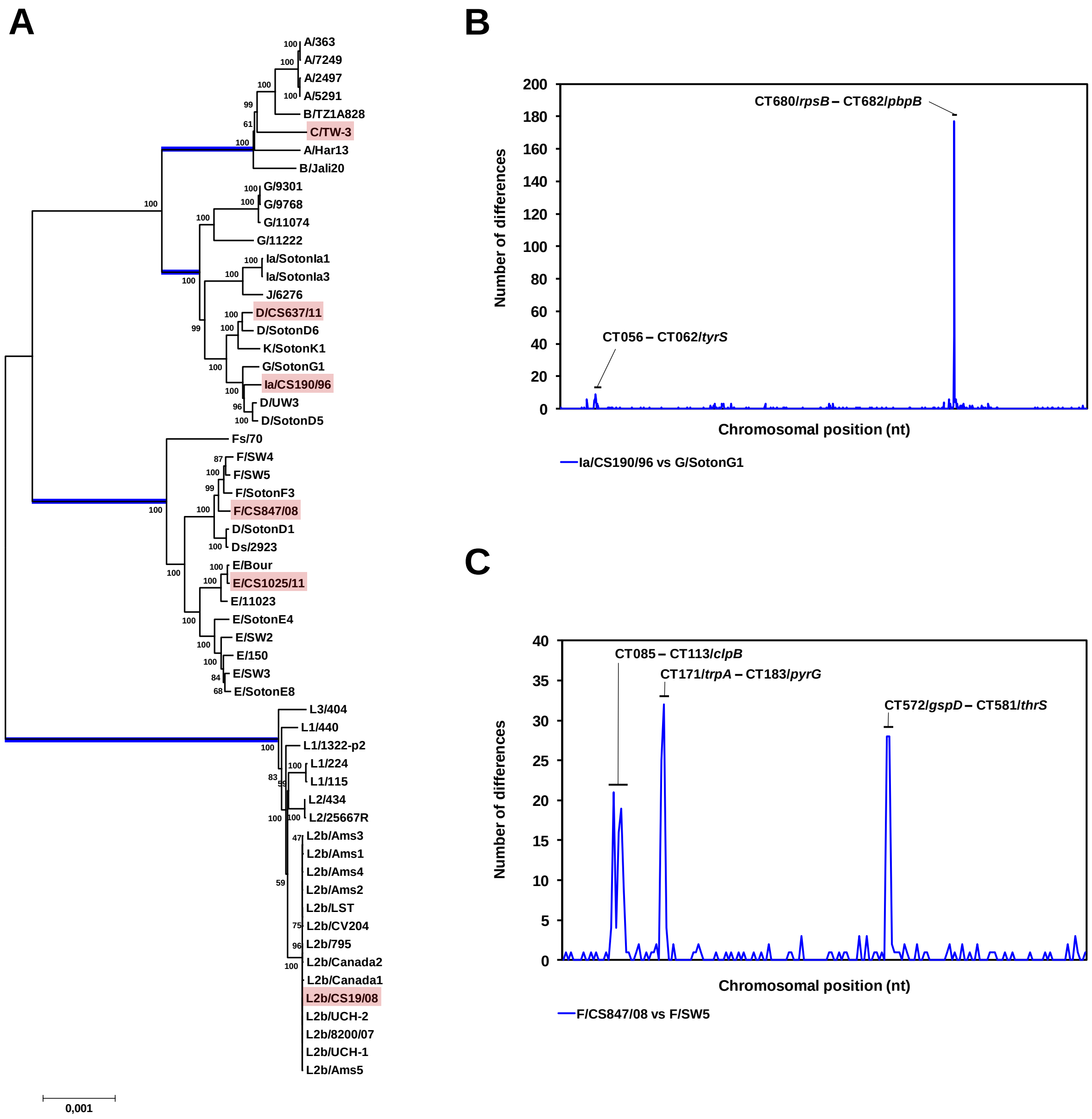

S1 Figure. Genome make-up of the studied *C. trachomatis* strains and evaluation of putative mosaic structures. Panel A. Evaluation of the genetic backbone of the studied strains in the frame of the species phylogeny and diversity. The phylogenetic tree was constructed (using MEGA5 [Tamura et al, 2011, Mol Biol Evol, 28:2731-2739]) based on a core-genome alignment enrolling 52 previously analyzed strains [Harris et al, 2012, Nat Genet, 44:413-419, S1] and the six strains evaluated in the present study (highlighted in red) by using the neighbor-joining method with bootstrapping (1000 replicates) [Saitou & Nei, 1987, Mol Biol Evol, 4:406-425] based on distance estimates using a Kimura two-parameter model for substitution events [Kimura, 1980, J Mol Evol, 16:111-120]. This analysis shows that the strains evaluated in the present experimental evolution study represent not only the three disease groups (ocular, epithelial-genital and LGV)

but also the four major genetic branches of the species tree in *C. trachomatis* (highlighted in blue): ocular clade (serovars A-C), prevalent epithelial-genital clade (mainly serovars E and F), non-prevalent epithelial-genital clade (serovars D, G, Ia, J, and K) and LGV clade (serovars L1-L3, and L2b). Panel B. Recombination analysis of the Ia/CS190/96 strain. The graph shows the SNP density across the chromosome when comparing the sequences from Ia/CS190/96 with G/SotonG1 (a probable parental strain). Polymorphism was assessed through DnaSP v5 analysis [Librado & Rozas, 2009, Bioinformatics, 25:1451-1452] using a window size and a step size of 1000 base pairs each. The Ia/CS190/96 is a recombinant strain likely resulting from the genetic import of the typing gene CT681/*ompA* and surrounding region (estimated crossovers occurring in the gene CT680/*rpsB* and in the IGR CT681/*ompA*-CT682/*pbpB*) from a serovar Ia parental strain to a strain with a similar genetic backbone of the serovar D or G strains clustering in the same sub-branch. The genetic backbone of the parental strain is likely more similar to that of G/SotonG1, as these two strains also share an unusual genetic feature at the plasmid (three and not four tandem 22-bp repeats at the ORI). The serovar Ia strain seems to have also imported a ~6000 bp region (enrolling the genes CT056 to CT062/*tyrS*) from a serovar E-like parental strain. Panel C. Recombination analysis of the F/CS847/08 strain. The graph shows the SNP density across the chromosome when comparing the sequences from the F/CS847/08 and F/SW5 strains. Polymorphism was assessed through DnaSP v5 analysis using a window size and a step size of 5000 base pairs each. The F/CS847/08 chromosome displays three putative recombining fragments enrolling the genomic regions from the genes: *i*) CT085 to CT113/*clpB* (with the deduced crossovers falling within the gene CT085 and a region spanning from the IGR CT107/*mutY* - CT108/*ybgI* to the CT113/*clpB* gene); *ii*) CT171/*trpA* to CT183/*pyrG* (putative crossovers occurring within the IGR CT171/*trpA*-CT172 and the gene CT183/*pyrG*); and *iii*) CT572/*gspD* to CT581/*thrS* (deduced crossovers likely occurred within a region enrolling half of the CT572/*gspD* gene and the 5'-end of the gene CT573, and within the CT581/*thrS* gene). The first two exchanged fragments were putatively inherited from a parental strain with a genetic backbone similar to the one from the strain E/Bour, whereas the third region matched the genome from a serovar G-like strain (closely related to the strains G/9301, G/9768 or G/11074).
