# Supplementary material for: Chlamydia trachomatis In Vivo to In Vitro Transition Reveals Mechanisms of Phase Variation and Down-Regulation of Virulence Factors
Source: PLoS One. 2015 Jul 24;10(7):e0133420. doi: 10.1371/journal.pone.0133420 (PMC4514472; doi:10.1371/journal.pone.0133420)
Supplement: S2 Fig — (PDF) [file pone.0133420.s002.pdf]

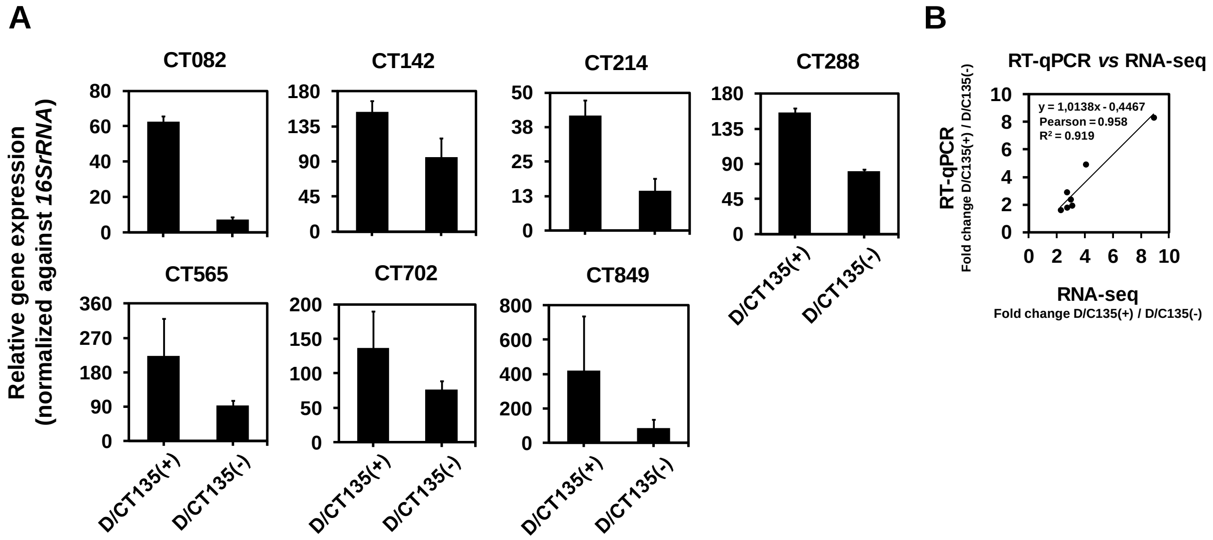

S2 Figure. Confirmation of the RNA-seq results with RT-qPCR. Panel A. RT-qPCR of seven differentially expressed genes that were chosen in order to represent a wide range of values regarding both expression levels and expression fold-change in the D/CS637/11 CT135-positive *versus* D/CS637/11 CT135-negative comparison. The expression values (mean ±SEM) resulted from raw RT-qPCR data (x10^5) of each gene normalized to that of the *16S rRNA*. Panel B. Correlation between the fold-change expression determined by RT-qPCR *versus* RNA-seq. The Pearson correlation coefficient (0.958), the curve slope (1.014) and the best fit linear regression analysis (R^2 = 0.919) demonstrate the high degree of correlation between the RT-qPCR and RNA-seq analyses.
