# Supplementary material for: Chlamydia trachomatis In Vivo to In Vitro Transition Reveals Mechanisms of Phase Variation and Down-Regulation of Virulence Factors
Source: PLoS One. 2015 Jul 24;10(7):e0133420. doi: 10.1371/journal.pone.0133420 (PMC4514472; doi:10.1371/journal.pone.0133420)
Supplement: S1 Table — (PDF) [file pone.0133420.s003.pdf]

S1 Table. Sequence data from the strains analyzed in this study.

Strain	Chromosome (bp) [a]	Plasmid (bp) [a]	Mean Depth Coverage Plasmid/Chromosome (ratio)					GenBank accession number [a]	
			P5-7	P20	P30	P50	P100	Chromosome	Plasmid
**C/TW-3**	1043554	7501	6717/1117 (6,01)	---	---	---	1823/262 (6,96)	CP006945	CP006946
**D/CS637/11**	1042680	7493	824/179 (4,60)	642/74 (8,68)	2318/266 (8,71)	---	---	CP007131	CP007132
**E/CS1025/11**	1043034	7502	6513/464 (14,03)	1520/104 (14,62)	2935/319 (9,20)	---	---	CP010567	CP010568
**F/CS847/08**	1043060	7493	3116/444 (7,02)	195/41 (4,76)	5727/1137 (5,04)	---	---	CP010569	CP010570
**Ia/CS190/96**	1042034	7471	4085/307 (13,31)	373/56 (6,66)	1164/139 (8,37)	3465/551 (6,29)	2107/247 (8,53)	CP010571	CP010572
**L2b/CS19/08**	1038864	7500	586/68 (8,62)	875/139 (6,29)	1258/104 (12,10)	---	---	CP009923	CP009924

[a] Both the sequence sizes and GenBank accession numbers refer to the chromosome and plasmid sequences of the major abundant clone in the first sequenced population.
