# Supplementary material for: Chlamydia trachomatis In Vivo to In Vitro Transition Reveals Mechanisms of Phase Variation and Down-Regulation of Virulence Factors
Source: PLoS One. 2015 Jul 24;10(7):e0133420. doi: 10.1371/journal.pone.0133420 (PMC4514472; doi:10.1371/journal.pone.0133420)
Supplement: S2 Table — (PDF) [file pone.0133420.s004.pdf]

S2 Table. Oligonucleotide primers used in PCR and qPCR assays.

ORF [a]	Primers	Primer sequence (5' to 3')	Primer location	Amplicon (bp)
RNA-seq vs RT-qPCR assays				
**CT082**	CT082-A	CAGCTCATGCAGCCAAAGAG	94352-94371	52
	CT082-B	GACGTTTCCGTAGCCTTGGAT	94383-94403	
**CT142**	CT142-A	ATGCTTGCGAAAGGTGTGTACA	159509-159530	51
	CT142-B	CCATCGCTTCCCAACTCCTA	159540-159559	
**CT214**	CT214-A	TTATTTCCGGACAAGCAGATGA	242770-242791	51
	CT214-B	TTTTGATCCCAATCCGATTAGG	242741-242762	
**CT288**	CT288-A	GCCTGCCTTTTATCGCTGTTAT	322619-322640	51
	CT288-B	CCATCCCCAATGCTAAGGAA	322650-322669	
**CT565**	CT565-A	CTATTACGCTAAAAACGGGCCTAT	636539-636562	51
	CT565-B	GCAACCCCGCATACCAAAG	636512-636530	
**CT702**	CT702-A	TTCTTCAGCCAGATTTGCTACTCA	807888-807911	52
	CT702-B	GCATGAATCAGCTTTTCCACATTA	807860-807833	
**CT849**	CT849-A	CAACAGCAATTAAACCAAGAAACG	998457-998480	51
	CT849-B	GCCAACGACAGCGTATTTGATT	998430-998451	
**CTr01-04**	*16SrRNA*-9 [c]	GCGAAGGCGCTTTTCTAATTTAT	854857-854879	76
	16*SrRNA*-10 [c]	CCAGGGTATCTAATCCTGTTTGCT	854909-854932	
mRNA decay assay (qPCR)				
**CT134**	CT134-A	CGGTTACGATGAGATTTGTTGTAGA	151694-151718	52
	CT134-B	CGCCACTACTTTTTCTGCAGTCT	151723-151745	
**CT135**	CT135-A	ACGAACGGATCATGTTTGAAGA	152387-152408	51
	CT135-B	CGGCTTCGAGAACACTAGGAACT	152415-152437	
Growth rate/doubling time and mRNA decay assays (qPCR)				
**CT681/*ompA***	OmpA-9 [c]	TGCCGCTTTGAGTTCTGCTT	780009-780028	76
	OmpA-10 [c]	GTCGATCATAAGGCTTGGTTCAG	779953-779975	

[a] Open reading frame (ORF) numbers are based on the D/UW3 strain genome annotation (GenBank No. NC_000117).
[b] Based on the chromosome sequence of D/UW3 strain (GenBank No. NC_000117).
[c] Previously described [Gomes et al, 2005, Microbes Infect, 7:410-420].
