# Supplementary material for: Chlamydia trachomatis In Vivo to In Vitro Transition Reveals Mechanisms of Phase Variation and Down-Regulation of Virulence Factors
Source: PLoS One. 2015 Jul 24;10(7):e0133420. doi: 10.1371/journal.pone.0133420 (PMC4514472; doi:10.1371/journal.pone.0133420)
Supplement: S3 Table — (PDF) [file pone.0133420.s005.pdf]

S3 Table. Genomic alterations throughout *in vitro* passaging.

Strain	Mutation location (chromosome) [a]	Mutation location (gene) [a]	*loci* [b]	nt change [c]	aa change [d]	Frequency (passage n.º)	Gene product / Putative role
**C/TW-3**							
	49197-49204	---	IGR CT043/*slc1*-CT044/*ssb*	DEL: 1 bp (AA → A) [e]	---	100% (P100)	Poly(A) tract in IGR upstream of CT043/*slc1* (chaperone of T3S effectors [98, 99]).
	153558	729	CT135	DEL: 1 bp (AA ↔ A)	Ψ	90%↔10% (P5-7); 100%↔0% (P100)	Putative Inc [33]; Virulence gene [46,47].
	191181	28	CTW3_00885	G → A	Val → Ile	100% (P100)	Cytotoxin remnant [72]
	213502	1634	CT189/*gyrA_1*	T → C	Phe → Ser	100% (P100)	DNA gyrase subunit A [77]
	764461	1345	CT664/*cdsD* or *yscD*	G → A	Gly → Ser	100% (P100)	FHA domains-containing protein predicted to form the inner membrane ring of T3S apparatus [100].
**D/CS637/11**							
	152763-154214	---	CT135 [f]	DEL: 1452 bp [f]	Ψ	100% (P20-P30)	Putative Inc [33]; Virulence gene [46,47].
	721797	760	CT633/*hemB*	G ↔ A	Ala ↔ Lys	80% ↔20% (P6); 0%↔100% (P20-P30)	Delta-aminolevulinic acid dehydratase [77].
	721798	761	CT633/*hemB*	C ↔ A	Ala ↔ Lys	80%↔20% (P6); 0%↔100% (P20-P30)	Delta-aminolevulinic acid dehydratase [77].
	825657	1021	CT713/*porB*	T → C	Ψ [g]	~30% (P30)	Porin [103].
**E/CS1025/11**							
	153379	443	CT135	DEL: 4 bp (AAATT → A)	Ψ	~40% (P20); ~100% (P30)	Putative Inc [33]; Virulence gene [46,47].
	602046-602057	---	IGR CT533/*lpxC* – CT534/*lnt*	DEL: 1bp (AA↔A) [e]	---	91%↔9% (P7); 77%↔23% (P20); 15%↔85% (P30)	Poly(A) tract upstream of CT533/*lpxC* (essential enzyme in the biosynthesis of lipid A) [75, 76].
	742284	2	CT645	T → C	Ψ [h]	~80% (P20); ~100% (P30)	Predicted integral membrane protein with unknown function (YGGT family) [77].
**F/CS847/08**							
	153373	543	CT135	C → A	Ψ	~50% (P20); ~59% (P30)	Putative Inc [33]; Virulence gene [46,47].
	153742	912	CT135	INS: 2 bp (A → AAA)	Ψ	~30% (P20); ~40% (P30)	Putative Inc [33]; Virulence gene [46,47].
	784264	2490	CT682/*pbpB*	G ↔ T	Leu ↔ Leu	37%↔63% (P7); 20%↔80% (P20); 44%↔56% (P30)	Penicillin-binding protein [77]; Synthesis of peptidoglycan.
	826262-826265	799-802	CT713/*porB*	INS: 1 bp (T → TT)	Ψ	~40% (P30)	Porin [103].
**Ia/CS190/96**							
	152948	315	CT135	DEL: 1 bp (AT → A)	Ψ	~28% (P30); 100% (P50-P100)	Putative Inc [33]; Virulence gene [46,47].
	230974	399	CT205/*pfkA*	T → G	Phe → Leu	~96% (P100)	Diphosphate--fructose-6-phosphate 1-phosphotransferase [77].
	289987	981	CT257	DEL: 1 bp (AA → A)	Ψ	100% (P50)	CBS domain-containing protein [96] suggested to display tropism for eukaryotic lipid-droplets [97].
	825140	978	CT713/*porB*	G → A	Ψ	~100% (P100)	Porin [103].
**L2b/CS19/08**							
	---	---	---	---	---	---	---

[a] Locations refer to the sequences (chromosome and gene) of the high frequent clone in the first population (passages 5-7), whose chromosome and plasmid sequences were deposited in GenBank (accession numbers are listed in S1 Table).

[b] The *loci* designations are based on genome annotation of the D/UW3 strain (GenBank accession number NC_000117) [77], except for the putative ORF CTW3_00885 (GenBank accession number CP006945) [72], which encodes a fragment of the ancestral *Chlamydia* cytotoxin predicted not to be functional [85]. IGR, intergenic region with adjacent ORFs indicated.

[c] The nucleotide changes in open reading frames are presented in the 5' to 3' direction. DEL, deletion. INS, insertion.

[d] Mutations leading to putative protein truncation are represented by the symbol Ψ.

[e] Homopolymeric poly(A) tract upstream of the locus coding sequence corresponding to poly(T) in the annotated leading strand.

[f] The mutation event involved the entire CT135 deletion.

[g] Lost of stop codon (TAA > CAA).

[h] Mutation in the start codon: ATG (Met) > ACG (Thr).
